# Supplementary material for: Immunotherapy targeting plasma ASM is protective in a mouse model of Alzheimer’s disease
Source: Nat Commun. 2023 Mar 24;14:1631. doi: 10.1038/s41467-023-37316-z (PMC10036484; doi:10.1038/s41467-023-37316-z)

Supplementary Information for

Immunotherapy targeting plasma ASM is protective in a mouse model of Alzheimer's disease

Byung Jo Choi,[1,2,11] Min Hee Park,[1,3,11] Kang Ho Park,[1,3] Wan Hui Han,[1,3] Hee Ji Yoon,[1,3] Hye Yoon Jung,[1,3] Ju Yeon Hong,[1,3] Md Riad Chowdhury,[1,3] Kyung Yeol Kim,[1,3] Jihoon Lee,[4] Im-Sook Song,[4] Minyeong Pang,[5] Min-Koo Choi,[5] Erich Gulbins,[6] Martin Reichel,[7] Johannes Kornhuber,[7] Chang-Won Hong,[3] Changho Kim,[8] Seung Hyun Kim,[9] Edward H. Schuchman,[10] Hee Kyung Jin,[1,2,*] Jae-sung Bae[1,3,*]

Correspondence to Jae-sung Bae, and Hee Kyung Jin
Email: jsbae@knu.ac.kr, and hkjin@knu.ac.kr

This PDF file includes:

Supplementary Figure 1 to 17
Supplementary Tables 1 to 2
Uncropped blots for Supplementary Figure

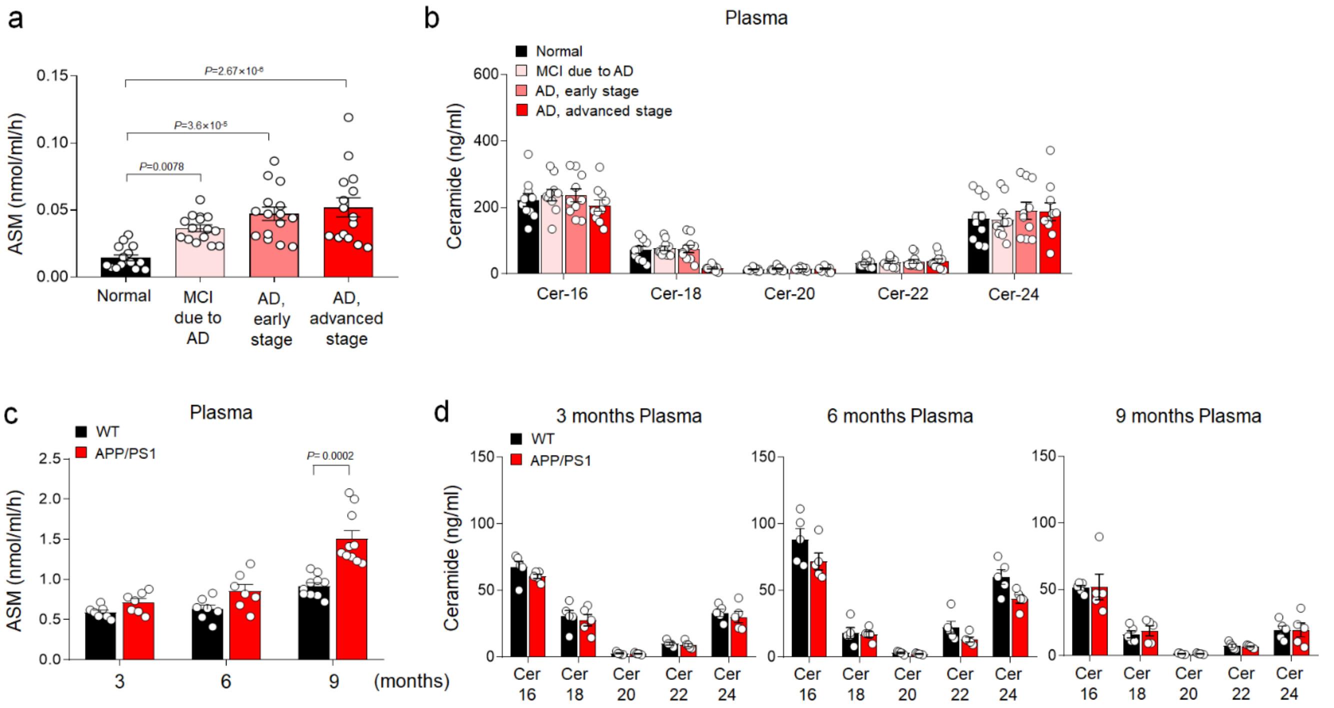

Supplementary Figure 1. ASM activity and ceramide levels in plasma or brain microvessels of AD patients or mice correlates with disease progression. a and b, ASM activity (**a**, n = 15/group) and ceramide concentration (**b**, n = 10/group) in plasma of normal and AD patients with disease progression. **c and d,** ASM activity (**c**, 3- and 6-month-old of WT and AD, n = 7; 9-month-old of WT and AD, n = 10) and ceramide concentration (**d**, n = 5/group) in plasma of 3-, 6-, and 9-month-old of WT and APP/PS1 mice. a, One-way analysis of variance, Tukey's post hoc test. c, Two-tailed student's t test. The data were collected from 3 independent experiments. All error bars indicate s.e.m. Source data are provided as a Source Data file.

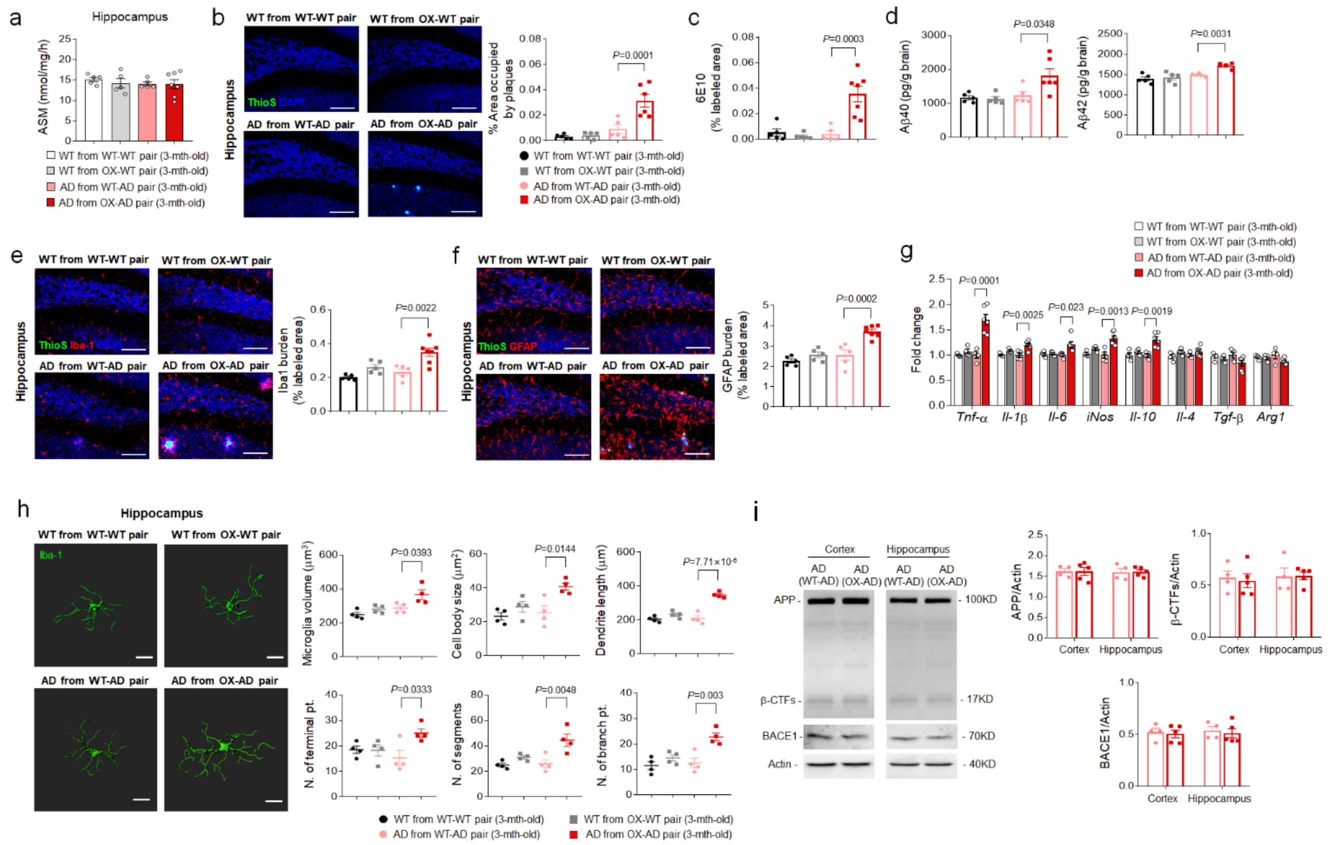

Supplementary Figure 2. Young APP/PS1 mice in parabiosis with *Smpd1*$^{ox/ox}$ mice show acceleration of Aβ deposits and microglia-mediated pro-inflammation in the hippocampus. a, ASM activity in the hippocampus (WT from WT-WT, WT from OX-WT, AD from WT-AD pair, n = 5; AD from OX-AD pair, n = 7). **b,** Representative immunofluorescence images and quantification of ThioS (Aβ plaques). Scale bars, 50 µm. (WT from WT-WT, WT from OX-WT, AD from WT-AD pair, n = 5; AD from OX-AD pair, n = 6). **c,** Quantification of 6E10 (WT from WT-WT, WT from OX-WT, AD from WT-AD pair, n = 5; AD from OX-AD pair, n = 7). **d**, Analysis of Aβ40 and Aβ42 depositions using ELISA kits (WT from WT-WT, WT from OX-WT, AD from WT-AD pair, n = 5; AD from OX-AD pair, n = 6). **e and f**, Immunofluorescence images and quantification of microglia (e, Iba-1, red) and astrocyte (f, GFAP, red) with Aβ plaques (ThioS, green) (WT from WT-WT, WT from OX-WT, AD from WT-AD pair, n = 5; AD from OX-AD pair, n = 7). Scale bars, 50 µm. **g**, mRNA levels of inflammatory markers in the hippocampus (WT from WT-WT, WT from OX-WT, AD from WT-AD pair, n = 4; AD from OX-AD pair, n = 6). **h**, Left, imaris-based three-dimensional images (Scale bars, 10 µm) of microglia. Right, imaris-based automated quantification of microglial morphology (n = 4/group). **i**, Western blot analysis and quantification of APP and β-CTF level and BACE1 level in the cortex and hippocampus (AD from WT-AD pair, n = 4; AD from OX-AD pair, n = 5). AD, APP/PS1; OX, *Smpd1*$^{ox/ox}$ (Tie2-cre; *Smpd1*$^{ox/ox}$ mice). b-h, One-way analysis of variance, Tukey's post hoc test. The data were collected from 3 independent experiments. All error bars indicate s.e.m. All data analysis was done at 4.5-mo-old mice. Source data are provided as a Source Data file.

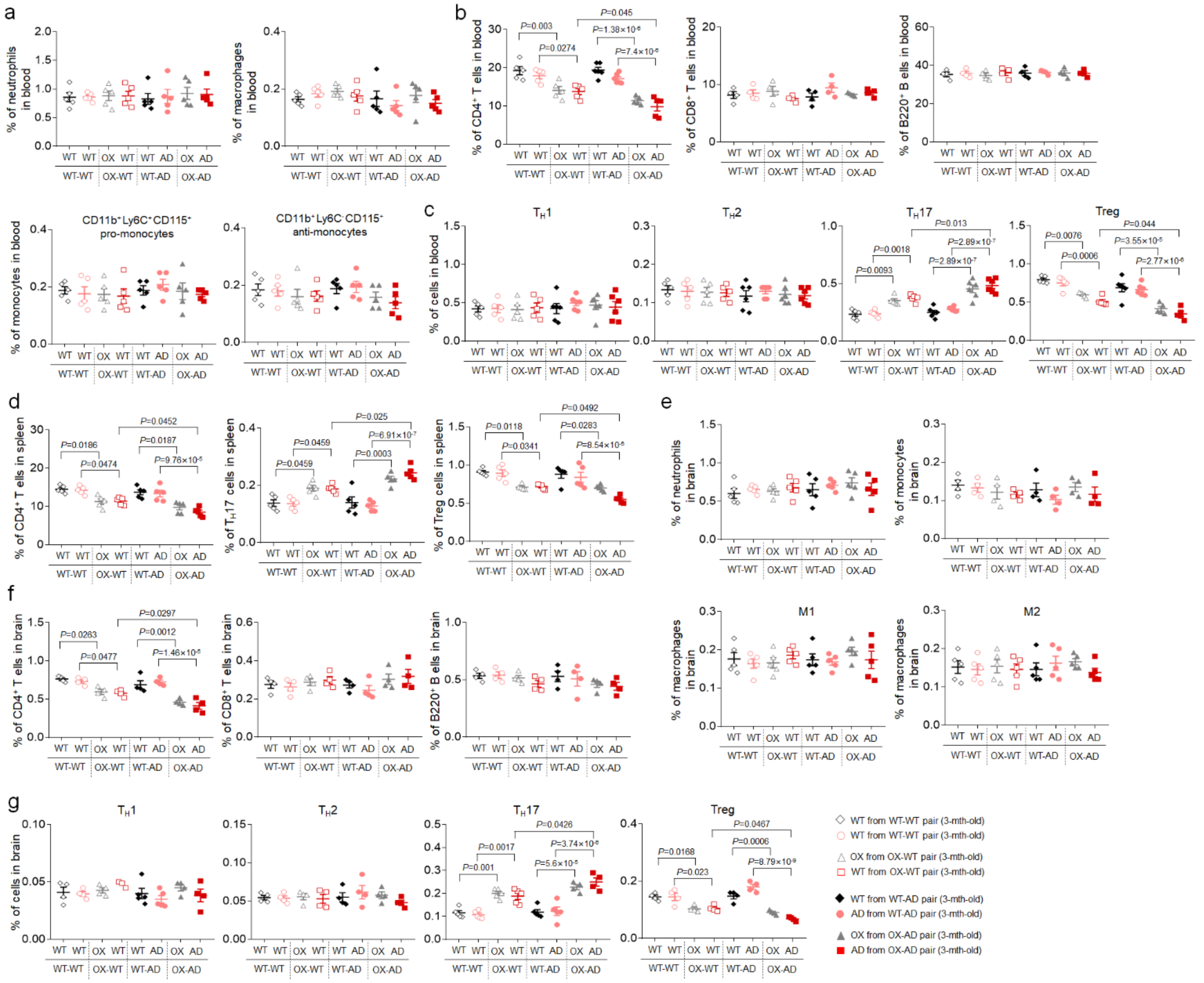

Supplementary Figure 3. Parabionts of young APP/PS1-*Smpd1*$^{ox/ox}$ mice exhibit reduction of CD4$^+$ T cells and Treg cells and increase of Th17 cells in the blood and brain. a, Percentage of neutrophils (CD11b$^+$ Ly6C$^+$ CD115$^-$), pro (CD11b$^+$ Ly6C$^+$ CD115$^+$)- or anti (CD11b$^+$ Ly6C$^-$ CD115$^+$)-monocytes, and macrophages (Lin$^-$ CD11b$^+$ F4/80^{hi} Ly6C$^-$) in the blood (n = 5/group). **b**, Percentage of CD4$^+$ T (WT and WT from WT-WT, OX and WT from OX-WT, OX and AD from OX-AD pair, n = 5; WT and AD from WT-AD pair, n = 6), CD8$^+$ T (n = 4/group), and B220$^+$ B cells (n = 4/group) in the blood. **c**, Percentage of Th1 (CD4$^+$ IFN-γ$^+$), Th2 (CD4$^+$ IL4$^+$), Th17 (CD4$^+$ IL17$^+$), and Treg (CD4$^+$ CD25$^+$ FOXP3$^+$) cells in the blood (Th1, Th2, Th17 cells: WT and WT from WT-WT, OX and WT from OX-WT pair, n = 5; WT and AD from WT-AD, OX and AD from OX-AD pair, n = 6, Treg cells: WT and WT from WT-WT, OX and WT from OX-WT, OX and AD from OX-AD pair, n = 5; WT and AD from WT-AD pair, n = 6). **d**, Percentage of CD4$^+$ T, Th17 (CD4$^+$ IL17$^+$), and Treg (CD4$^+$ CD25$^+$ FOXP3$^+$) cells in the spleen (n = 5/group). **e**, Percentage of neutrophils (CD45^{hi} CD11b$^+$ Gr-1$^+$), monocytes (CD45^{hi} CD11b$^+$ Ly6C^{hi} Ly6G^{low}), and M1 (CD45^{hi} CD11b$^+$ Ly6G$^-$ F4/80$^+$ MHCII^{hi})- or M2 (CD45^{hi} CD11b$^+$ Ly6G$^-$ F4/80$^+$ CD206$^+$)-macrophages in the brain (neutrophils, M1- or M2 macrophages: n = 5/group; monocytes: n = 4/group). **f**, Percentage of CD4$^+$ T, CD8$^+$ T, and B220$^+$ B cells in the brain (n = 4/group). **g**, Percentage of Th1 (CD4$^+$ IFN-γ$^+$), Th2 (CD4$^+$ IL4$^+$), Th17 (CD4$^+$ IL17$^+$), and Treg (CD4$^+$ CD25$^+$ FOXP3$^+$) cells in the

brain (Th1: WT and WT from WT-WT, OX and WT from OX-WT, OX and AD from OX-AD pair, n = 4; WT and AD from WT-AD pair, n = 5; Th2, Treg cells: n = 4/group; Th17 cells: WT and WT from WT-WT, OX and WT from OX-WT, WT and AD from WT-AD pair, n = 5; OX and AD from OX-AD pair, n = 4). AD, APP/PS1; OX, *Smpd1*$^{ox/ox}$ (Tie2-cre; *Smpd1*$^{ox/ox}$ mice). b-d, f, g, One-way analysis of variance, Tukey's post hoc test or two-tailed student's t test. The data were collected from 3 independent experiments. All error bars indicate s.e.m. All data analysis was done at 4.5-mo-old mice. Source data are provided as a Source Data file.

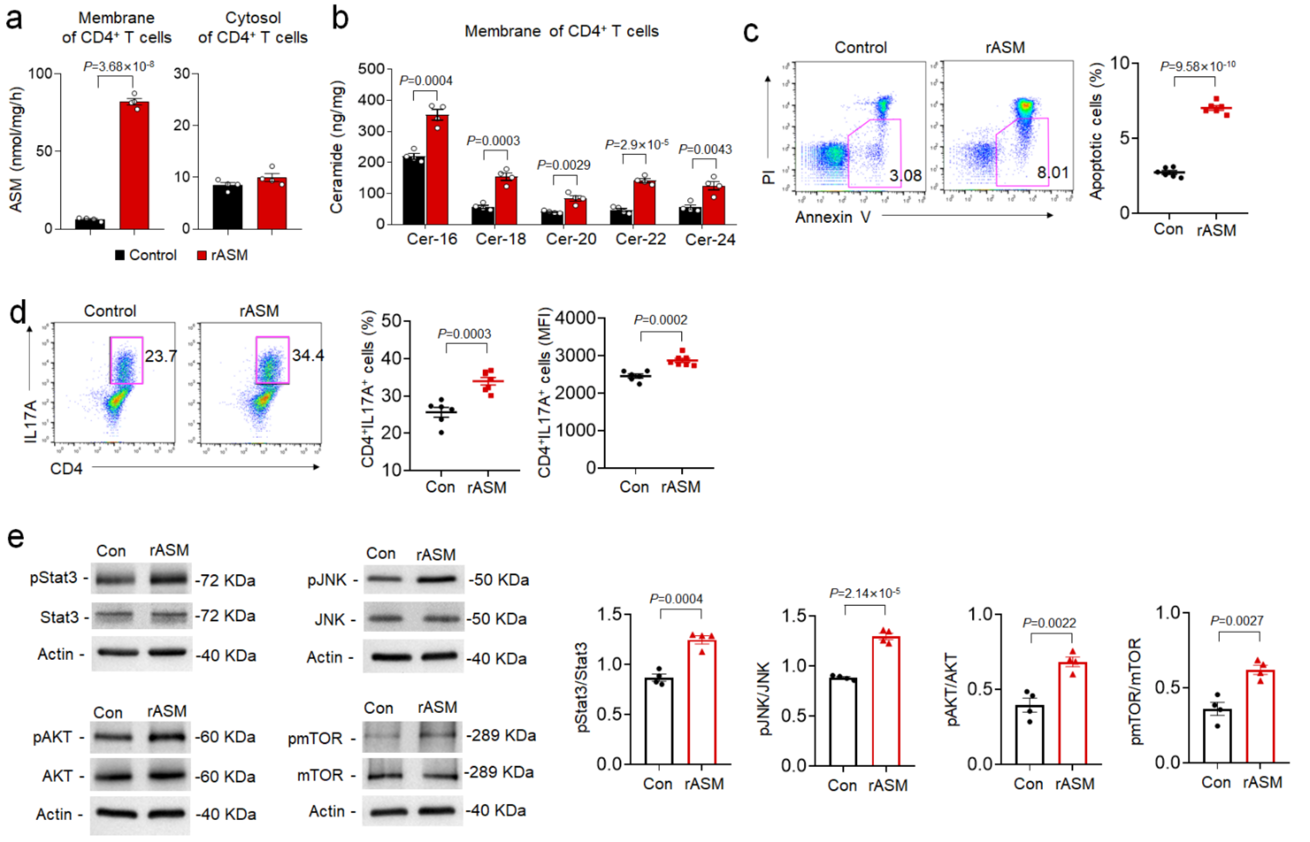

Supplementary Figure 4. Recombinant human ASM induces CD4$^+$ T cell apoptosis and pathogenic Th17 differentiation by increasing ceramide in cell membranes. a, ASM activity in membrane and cytosol of CD4$^+$ T cells treated with or without rASM (n = 4/group). **b**, Ceramide concentration in membrane of CD4$^+$ T cells (n = 4/group). **c**, Percentage of apoptotic cells detected with Annexin V$^+$ in CD4$^+$ T cells (n = 6/group). **d**, Representative flow cytometry plot and graph displaying the calculated percentage and mean fluorescent intensity of Th17 cells differentiated from CD4$^+$ T cells (Control, n = 6; rASM, n = 7). **e**, Western blotting for p-Stat3, p-JNK, p-AKT, and p-mTOR in Th17 cells differentiated from CD4$^+$ T cell by rASM treatment (n = 4/group). a-e, Two-tailed student's t test. The data were collected from one independent experiments. All error bars indicate s.e.m. Source data are provided as a Source Data file.

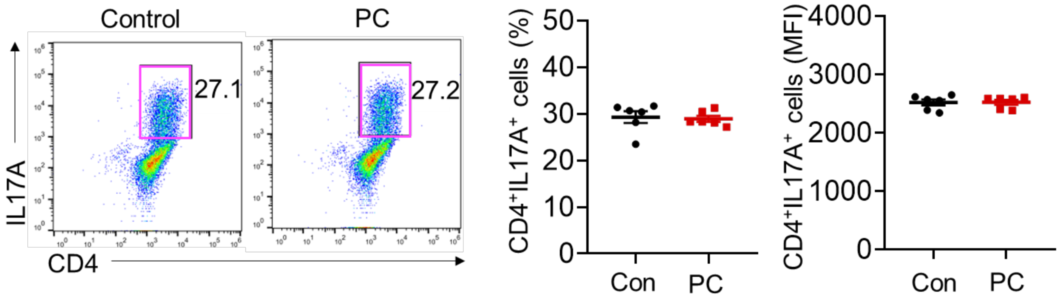

Supplementary Figure 5. Phosphorylcholine does not induce Th17 cell differentiation from $CD4^+$ T cells. Representative flow cytometry plot and graph displaying the calculated percentage and mean fluorescent intensity of Th17 cells differentiated from $CD4^+$ T cells treated with or without phosphorylcholine (n = 6/group). The data were collected from 3 independent experiments. All error bars indicate s.e.m. Source data are provided as a Source Data file.

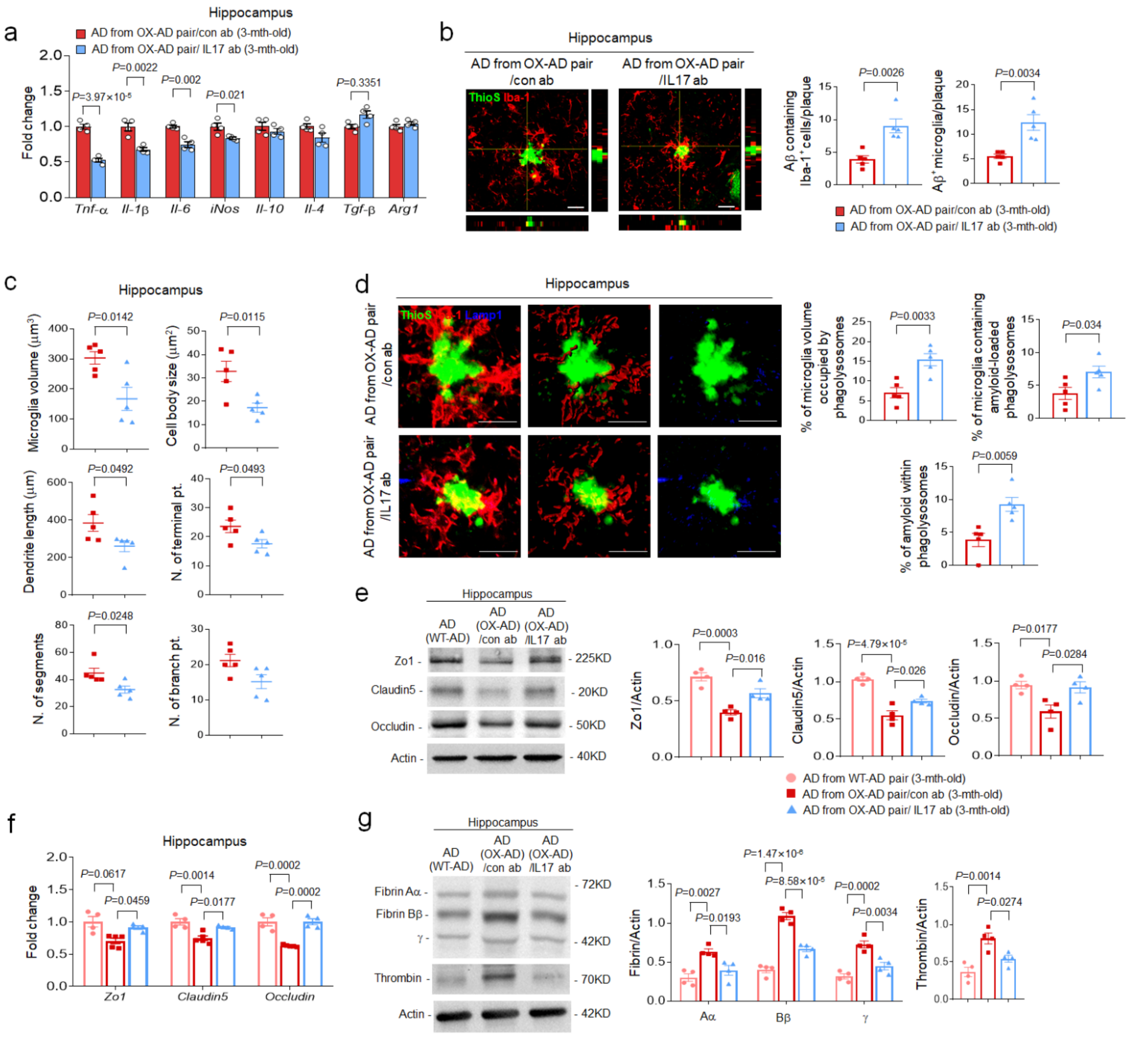

Supplementary Figure 6. Inhibition of pathogenic Th17 cells prevents microglia phagocytic function deficiency and BBB disruption in the hippocampus of young APP/PS1 mice in parabiosis with *Smpd1*$^{ox/ox}$ mice. a, mRNA levels of inflammatory markers in the hippocampus (n = 4/group). **b**, Immunostaining images of the colocalization of microglia (Iba1, red) with Aβ aggregates (ThioS, green) and quantification of Aβ positive cells and microglia. (n = 5/group). Scale bars = 10 μm. **c**, Imaris-based automated quantification of microglial morphology (n = 5/group). **d**, Left, immunofluorescence images of ThioS (Aβ plaques, green) encapsulated within Lamp1^{+} structures (phagolysosomes, blue) in microglia (Iba1, red) present in the hippocampus of each group. Scale bars, 20 μm; 3D reconstruction from confocal image stacks scale bars, 10 μm. Right, quantification of microglia volume occupied by Lamp1^{+} phagolysosomes, percent of microglia containing Aβ-loaded phagolysosomes and Aβ encapsulated in phagolysosomes (n = 5/group). **e**, Western blot analysis and quantification of tight junction proteins (Zo1, Claudin5, Occludin) in the hippocampus (n = 4/group). **f**, mRNA levels of tight junction (AD from WT-AD pair, AD from OX-AD pair/IL17 ab, n = 4; AD from OX-AD pair/con ab, n = 5). **g**, Western blot

analysis and quantification of fibrin and thrombin (n = 4/group). AD, APP/PS1; OX, *Smpd1*$^{ox/ox}$ (Tie2-cre; *Smpd1*$^{ox/ox}$ mice). a-d, Two-tailed student's t test. e-g, One-way analysis of variance, Tukey's post hoc test. All error bars indicate s.e.m. The data were collected from 3 independent experiments. All data analysis was done at 4.5-mo-old mice. Source data are provided as a Source Data file.

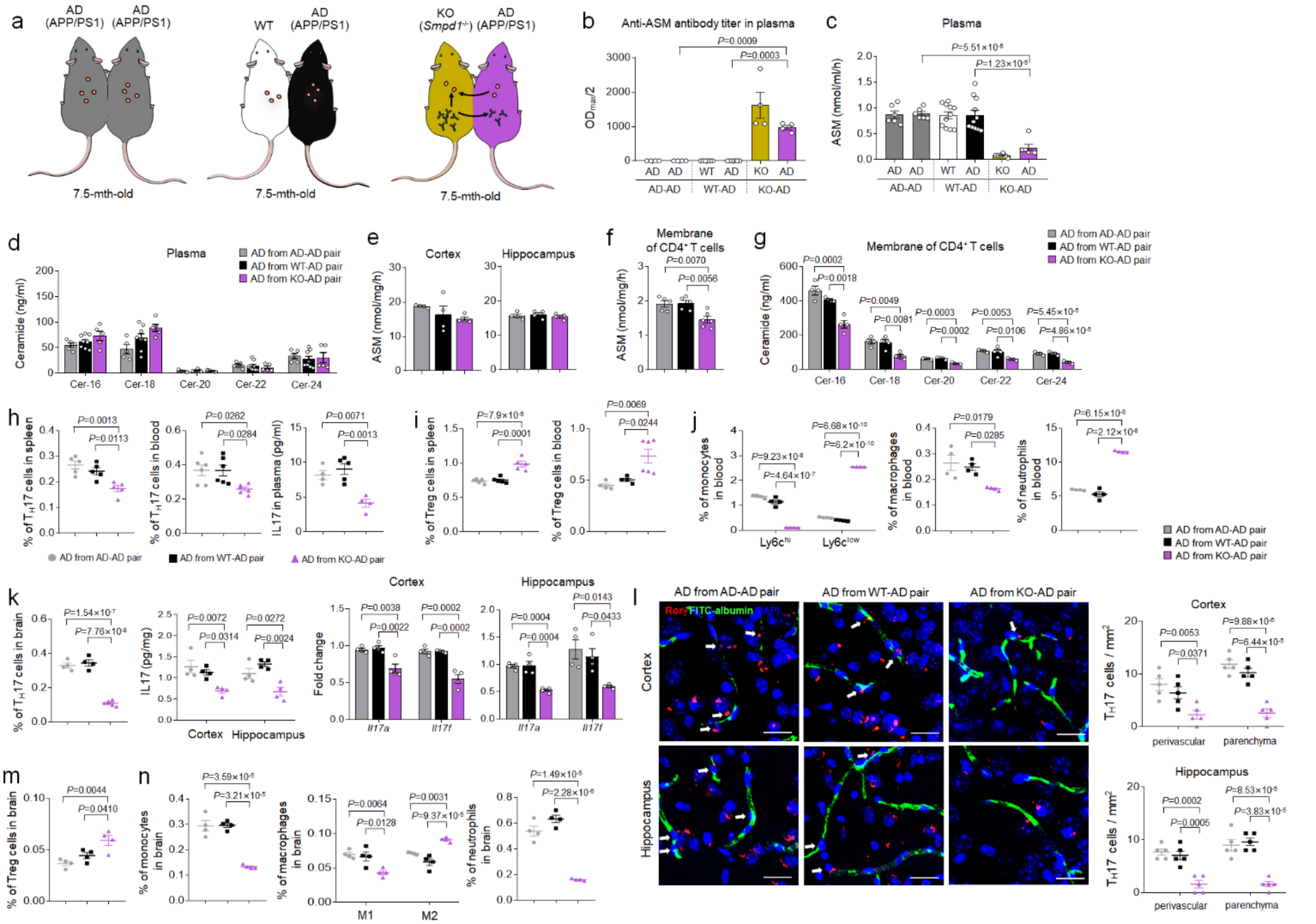

Supplementary Figure 7. Antibody-based inhibition of plasma ASM activity reduces pathogenic Th17 cells and pro-inflammatory myeloid immune cells in the blood and brain of APP/PS1 mice in parabiosis with *Smpd1*$^{-/-}$ mice. a, Schematic showing parabiotic pairings. **b**, Anti-ASM antibody titers in plasma of each parabiont (AD and AD from AD-AD, KO and AD from KO-AD pair, n = 4; WT and AD from WT-AD pair, n = 6). **c and d**, ASM activity (**c**, AD and AD from AD-AD, KO and AD from KO-AD pair, n = 6; WT and AD from WT-AD pair, n = 11) and ceramide concentrations (**d**, AD from AD-AD, AD from KO-AD pair, n = 5; AD from WT-AD pair, n = 8) in plasma. **e**, ASM activity in cortex and hippocampus (n = 4/group). **f and g**, ASM activity (**f**, AD from AD-AD, AD from WT-AD pair, n = 5; AD from KO-AD pair, n = 6) and ceramide levels (**g**, n = 4/group) in membrane of CD4^{+} T cell isolated from APP/PS1 mice exposed with blood of APP/PS1, WT, or *Smpd1*$^{-/-}$ mice. **h**, Percentage of Th17 cells in the spleen (n = 5/group) and blood (n = 6/group) and protein levels of IL17 in plasma (AD from AD-AD, AD from KO-AD pair, n = 4; AD from WT-AD pair, n = 5). **i**, Percentage of Treg cells in the spleen (n = 5/group) and blood (AD from AD-AD, AD from WT-AD pair, n = 4; AD from KO-AD pair, n = 6). **j**, Percentage of pro (Ly6c^{hi})- or anti (Ly6c^{low})-monocytes, macrophages, and neutrophils in the blood (n = 4/group). **k**, Percentage of Th17 cells in the brain (AD from AD-AD, AD from WT-AD pair, n = 4; AD from KO-AD pair, n = 6), protein levels of IL17, and mRNA levels of *Il17a* and *Il17f* in the cortex and hippocampus (n = 4/group). **l**, Immunofluorescence images and quantification of Th17 cells (RORγ, red) in the perivascular (blood vessel, FITC-labeled albumin, green) and parenchyma cortex and hippocampus regions (n = 5/group). Scale bars, 20 μm. White arrow: perivascular Th17 cells. Extravascular cells were counted as parenchymal Th17 cells. **m**, Percentage of Treg cells in the brain (n = 4/group). **n**, Percentage

of monocytes, M1 or M2 macrophages, and neutrophils in the brain (n = 4/group). All immune cells were analyzed by flow cytometry. AD, APP/PS1; KO, *Smpd1*$^{-/-}$. b-n, One-way analysis of variance, Tukey's post hoc test. The data were collected from 3 independent experiments. All error bars indicate s.e.m. All data analysis was done at 9-mo-old mice. Source data are provided as a Source Data file.

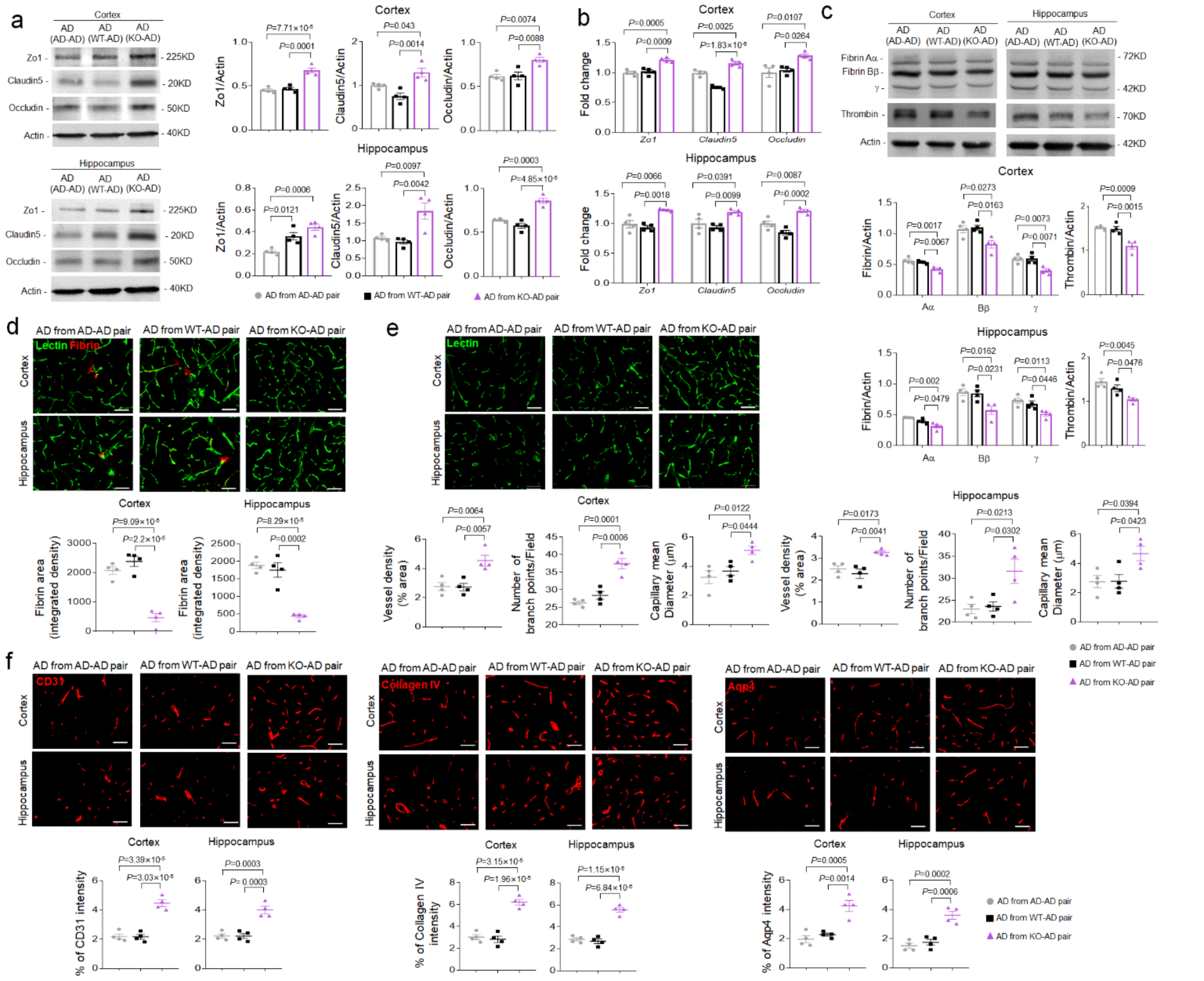

Supplementary Figure 8. Antibody-based inhibition of plasma ASM activity protects BBB disruption of APP/PS1 mice. a, Western blot analysis and quantification of tight junction proteins (Zo1, Claudin5, Occludin) in the cortex and hippocampus (n = 4/group). **b**, mRNA levels of tight junction (n = 4/group). **c**, Western blot analysis and quantification of fibrin and thrombin (n = 4/group). **d**, Top, immunofluorescence images of extravascular fibrin deposits in cortex and hippocampus. Scale bars, 50 μm. Bottom, quantification of extravascular fibrin deposits (n = 4/group). **e**, Representative images and quantification of lectin-positive microvascular profiles in cortex and hippocampus (n = 4/group). Scale bars, 50 μm. **f**, Representative image and quantification of CD31, collagen IV, and aquaporin-4 in cortex and hippocampus (n = 4/group). Scale bar: 50μm. AD, APP/PS1; KO, *Smpd1*$^{-/-}$. a-f, One-way analysis of variance, Tukey's post hoc test. The data were collected from 3 independent experiments. All error bars indicate s.e.m. All data analysis was done at 9-mo-old mice. Source data are provided as a Source Data file.

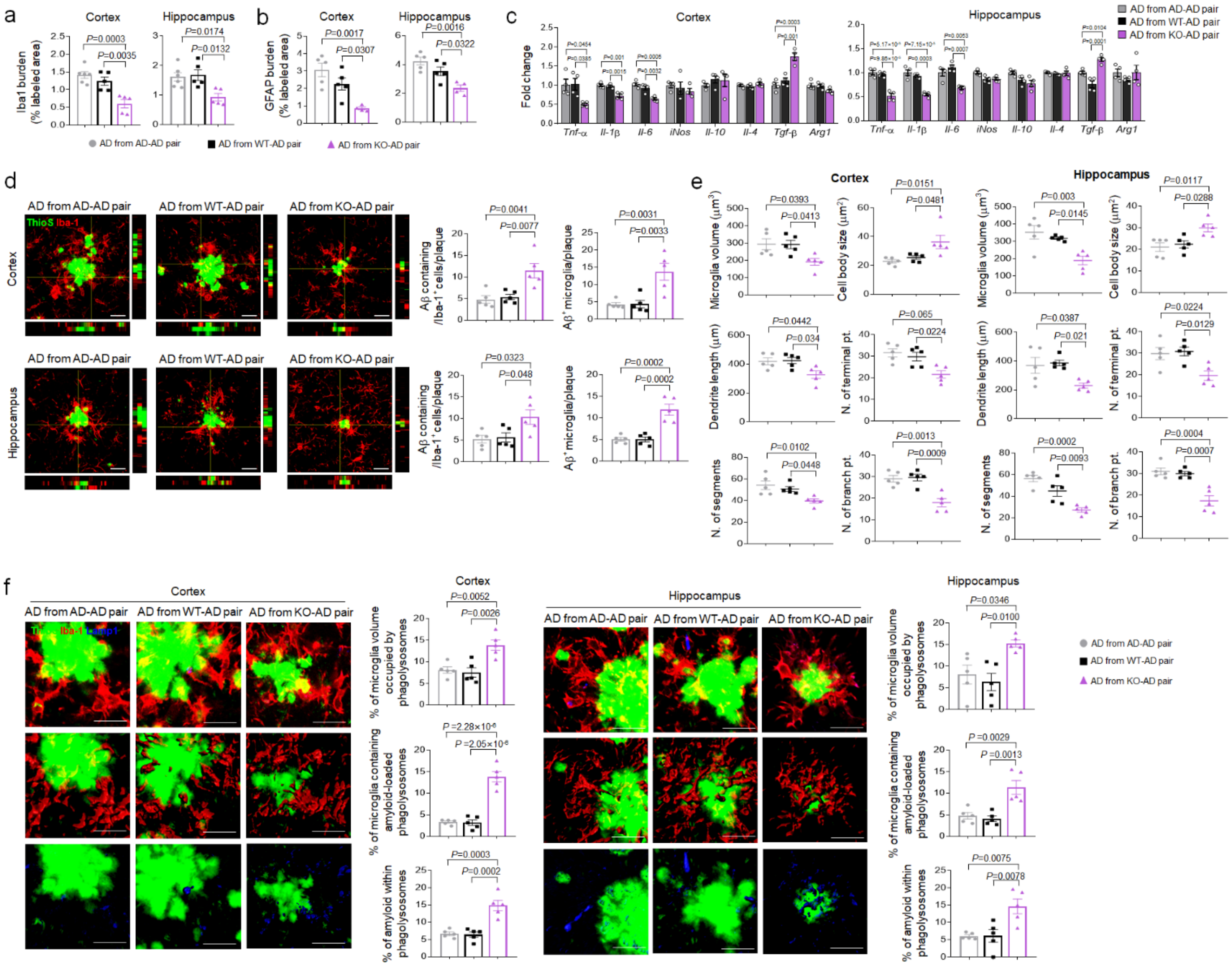

Supplementary Figure 9. Antibody-based inhibition of plasma ASM activity prevents neuroinflammation and deficiency of microglia phagocytic function in APP/PS1 mice. a and b, Quantification of microglia (a, Iba-1, AD from AD-AD pair, n = 6; AD from WT-AD, AD from KO-AD pair, n = 5) and astrocyte (b, GFAP, AD from AD-AD, AD from WT-AD pair, n = 5; AD from KO-AD pair, n = 4). **c**, mRNA levels of inflammatory markers in the cortex and hippocampus (n = 4/group). **d**, Immunostaining images of the colocalization of microglia (Iba1, red) with Aβ aggregates (ThioS, green) and quantification of Aβ positive cells and microglia. (n = 5/group). Scale bars = 10 μm. **e**, Imaris-based automated quantification of microglial morphology (n = 5/group). **f**, Immunofluorescence images of ThioS (Aβ plaques, green) encapsulated within Lamp1^{+} structures (phagolysosomes, blue) in microglia (Iba1, red) present in cortex and hippocampus of each group. Scale bars, 20 μm; 3D reconstruction from confocal image stacks scale bars, 10 μm. Quantification of microglia volume occupied by Lamp1^{+} phagolysosomes, percent of microglia containing Aβ-loaded phagolysosomes and Aβ encapsulated in phagolysosomes (n = 5/group). AD, APP/PS1; KO, *Smpd1*$^{-/-}$. a-f, One-way analysis of variance, Tukey's post hoc test. The data were collected from 3 independent experiments. All error bars indicate s.e.m. All data analysis was done at 9-mo-old mice. Source data are provided as a Source Data file.

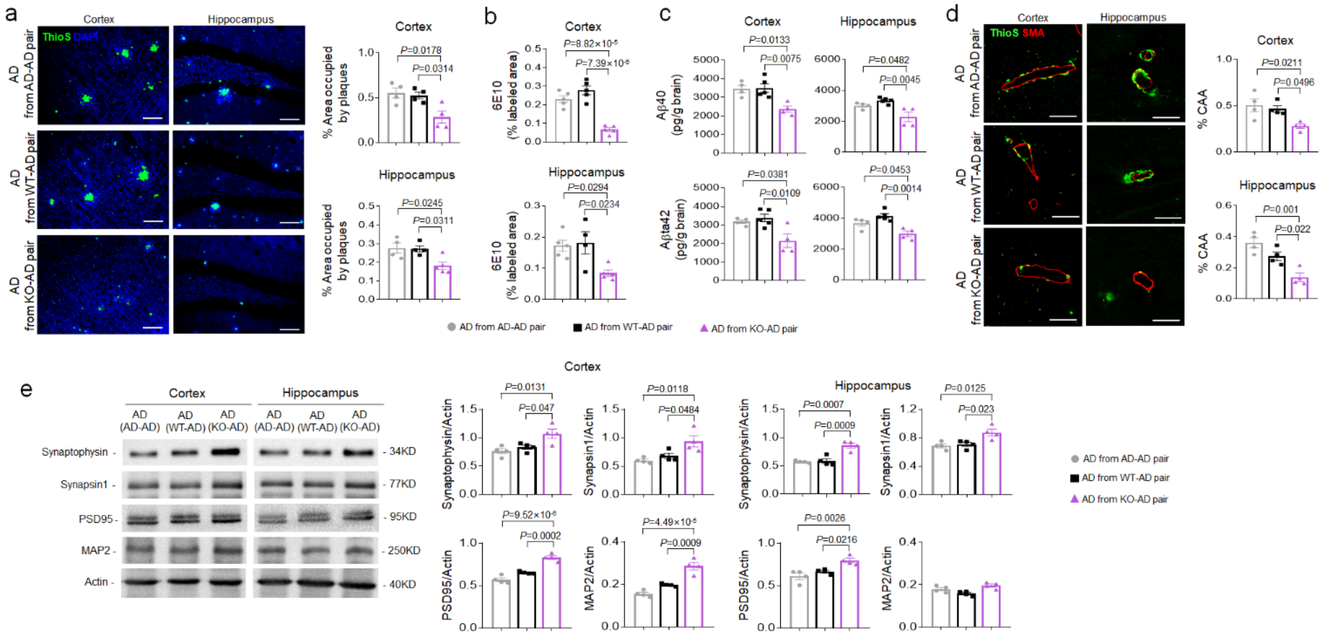

Supplementary Figure 10. Antibody-based inhibition of plasma ASM activity reduces Aβ accumulation and synapse loss in APP/PS1 mice. a, Left, representative immunofluorescence images of thioflavin S (ThioS, Aβ plaques) in cortex (n = 4/group) and hippocampus (AD from AD-AD, AD from WT-AD pair, n = 4; AD from KO-AD pair, n = 5). Scale bars, 50 µm. Right, quantification of area occupied by Aβ plaques. **b,** Quantification of 6E10 (cortex: n = 5/group; hippocampus: AD from AD-AD, AD from KO-AD pair, n = 5; AD from WT-AD pair, n = 4). **c**, Analysis of Aβ40 and Aβ42 depositions using ELISA kits (AD from AD-AD, AD from KO-AD pair, n = 4; AD from WT-AD pair, n = 5). **d**, Representative images and quantification of amyloid angiopathy (n = 4/group, CAA). To confirm amyloid angiopathy, brain section was stained with Aβ plaque (ThioS, green) and smooth muscle actin for vascular smooth muscle cells (SMA, red). Scale bars, 20 µm. **e**, Western blot analysis for synaptophysin, synapsin1, PSD95, and MAP2 in each group (n = 4/group). AD, APP/PS1; KO, *Smpd1*$^{-/-}$. a-e, One-way analysis of variance, Tukey's post hoc test. The data were collected from 3 independent experiments. All error bars indicate s.e.m. All data analysis was done at 9-mo-old mice. Source data are provided as a Source Data file.

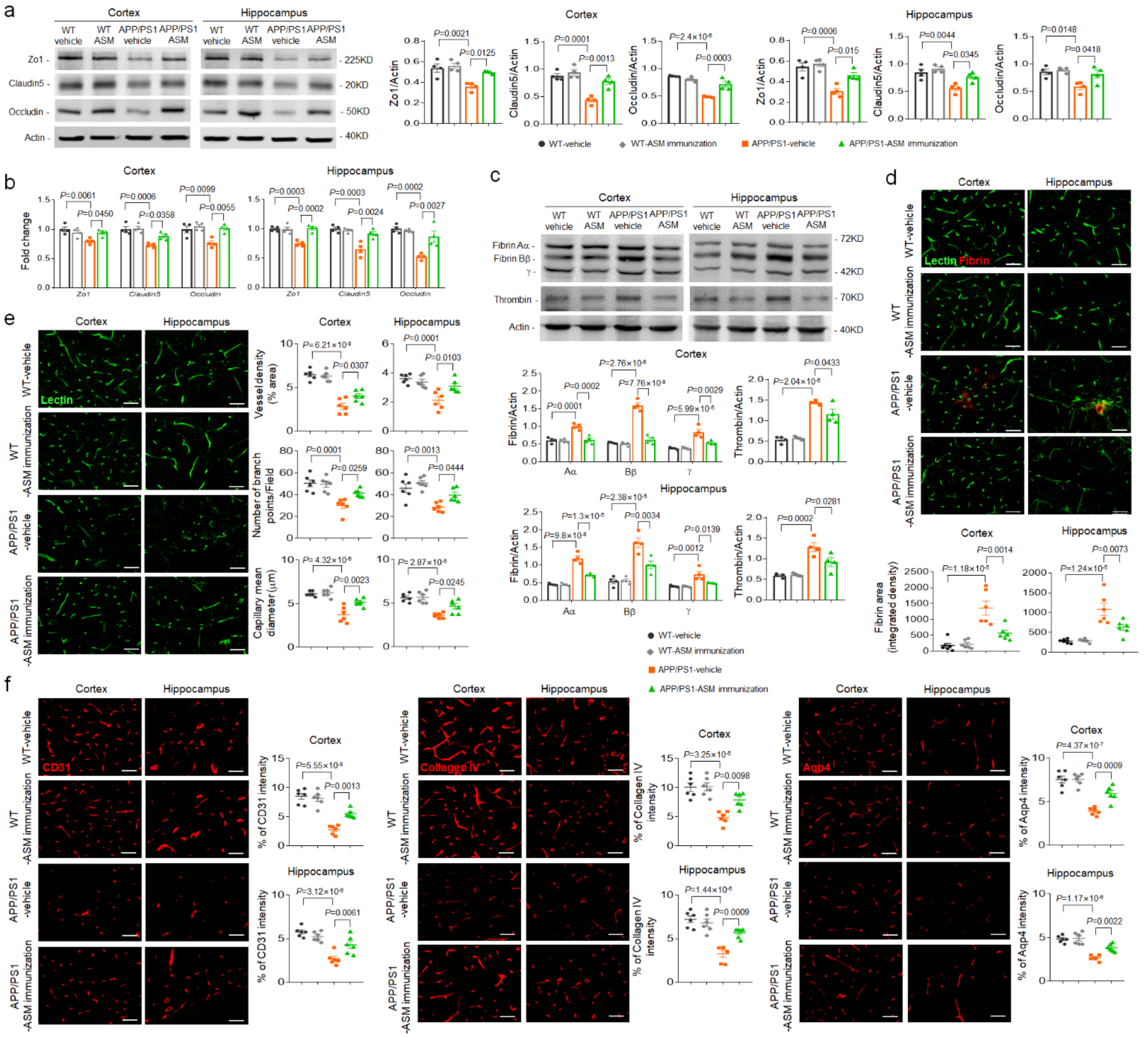

Supplementary Figure 11. Immunization with an ASM peptide protects BBB disruption in APP/PS1 mice. a, Western blot analysis and quantification of tight junction proteins (Zo1, Claudin5, Occludin) in the cortex and hippocampus (n = 4/group). **b**, mRNA levels of tight junction (n = 4/group). **c**, Western blot analysis and quantification of fibrin and thrombin (n = 4/group). **d**, Top, immunofluorescence images of extravascular fibrin deposits in cortex and hippocampus. Scale bars, 50 µm. Bottom, quantification of extravascular fibrin deposits (n = 6/group). **e**, Representative images and quantification of Lectin-positive microvascular profiles in cortex and hippocampus (n = 6/group). Scale bars, 50 µm. **f**, Representative image and quantification of CD31, collagen IV, and aquaporin-4 in cortex and hippocampus (n = 6/group). Scale bar: 50µm. a-f, One-way analysis of variance, Tukey's post hoc test. The data were collected from 3 independent experiments. All error bars indicate s.e.m. All data analysis was done at 9-mo-old mice. Source data are provided as a Source Data file.

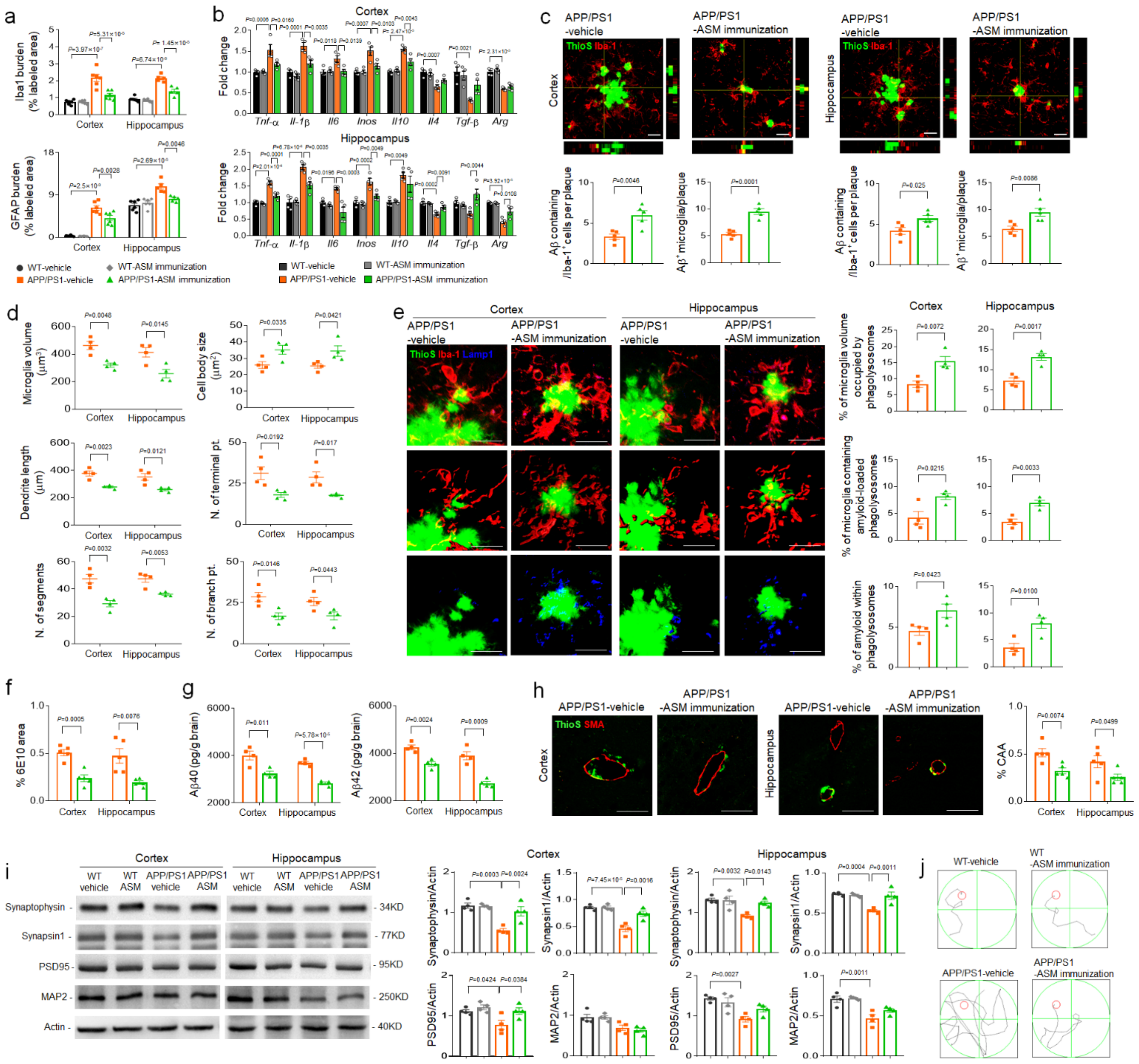

Supplementary Figure 12. Immunization with an ASM peptide prevents neuroinflammation, impairment of microglia phagocytic function, Aβ accumulation, and synapse loss in APP/PS1 mice. **a**, Quantification of microglia (Iba-1, cortex: n = 6/group; hippocampus: WT-vehicle, WT-ASM, n = 6; APP/PS1-vehicle, APP/PS1-ASM, n= 5) and astrocyte (GFAP, cortex: n = 6/group; hippocampus: WT-vehicle, WT-ASM, n = 6; APP/PS1-vehicle, APP/PS1-ASM, n= 5). **b**, mRNA levels of inflammatory markers in the cortex and hippocampus (n = 4/group). **c**, Immunostaining images of the colocalization of microglia (Iba1, red) with Aβ aggregates (ThioS, green) and quantification of Aβ positive cells and microglia. (n = 5/group). Scale bars = 10 μm. **d**, Imaris-based automated quantification of microglial morphology (n = 4/group). **e**, Immunofluorescence images of ThioS (Aβ plaques, green) encapsulated within Lamp1^{+} structures (phagolysosomes, blue) in microglia (Iba1, red) present in cortex and hippocampus of each group. Scale bars, 20 μm; 3D reconstruction from confocal image stacks scale bars, 10 μm. Quantification of microglia volume occupied by Lamp1^{+} phagolysosomes, percent of microglia

containing Aβ-loaded phagolysosomes and Aβ encapsulated in phagolysosomes (n = 4/group). **f,** Quantification of 6E10 (n = 5/group). **g**, Analysis of Aβ40 and Aβ42 depositions using ELISA kits (n = 4/group). **h**, Representative images and quantification of amyloid angiopathy (n = 5/group, CAA). To confirm amyloid angiopathy, brain section was stained with Aβ plaque (ThioS, green) and smooth muscle actin for vascular smooth muscle cells (SMA, red). Scale bars, 20 μm. **i**, Western blot analysis for synaptophysin, synapsin1, PSD95, and MAP2 in each group (n = 4/group). **j**, Representative swimming paths at day 10 of training of morris water maze test. a,b,i, One-way analysis of variance, Tukey's post hoc test. c-h, Two-tailed student's t test. The data were collected from 3 independent experiments. All error bars indicate s.e.m. All data analysis was done at 9-mo-old mice. Source data are provided as a Source Data file.

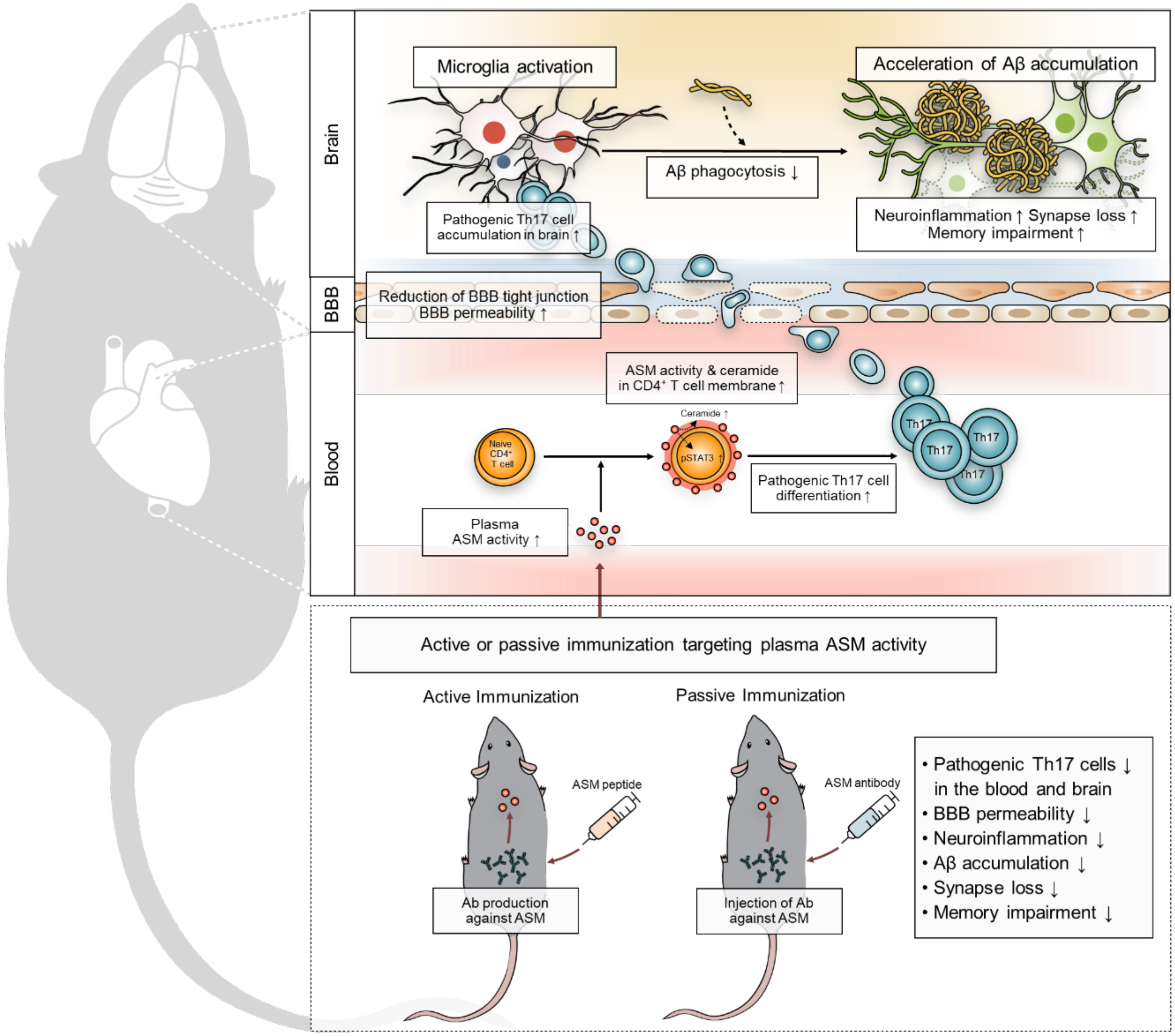

Supplementary Figure 13. The prophylactic effects of active or passive immunization targeting plasma ASM activity on neuropathological features in AD mice. Increase of plasma ASM activity causes differentiation of pathogenic Th17 cells by elevating ceramide in $CD4^+$ T cell membrane of spleen and blood. Pathogenic Th17 cells promote BBB disruption by reducing tight junction expression on endothelial cells, contributing to increase of BBB permeability. The BBB breakdown facilitates the entrance in the brain parenchymal of pathogenic Th17 cells. Consequently, pathogenic Th17 cells infiltrated in the brain impacts on microglia activation and impaired phagocytosis functions, resulting to acceleration of Aβ accumulation and further neuroinflammation, synapse loss, and memory impairment. The antibody-based immunotherapy targeting plasma ASM through active or passive immunization shows efficient inhibition of ASM activity in the blood. Interestingly, it leads to prophylactic effects on theses neuropathological features by suppressing pathogenic Th17 cells in AD mice.

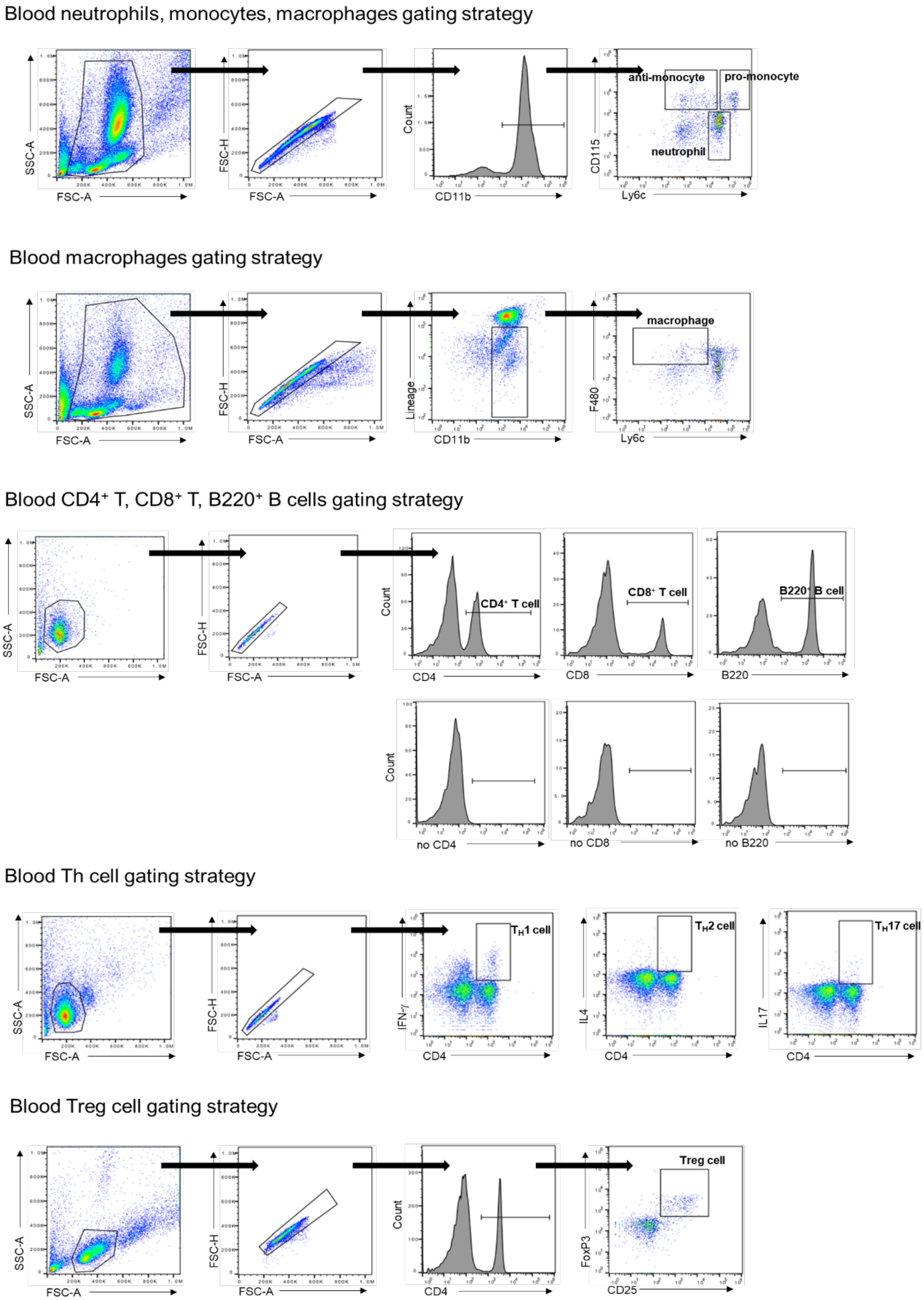

Supplementary Figure 14. Blood immune cells FACs gating strategy.

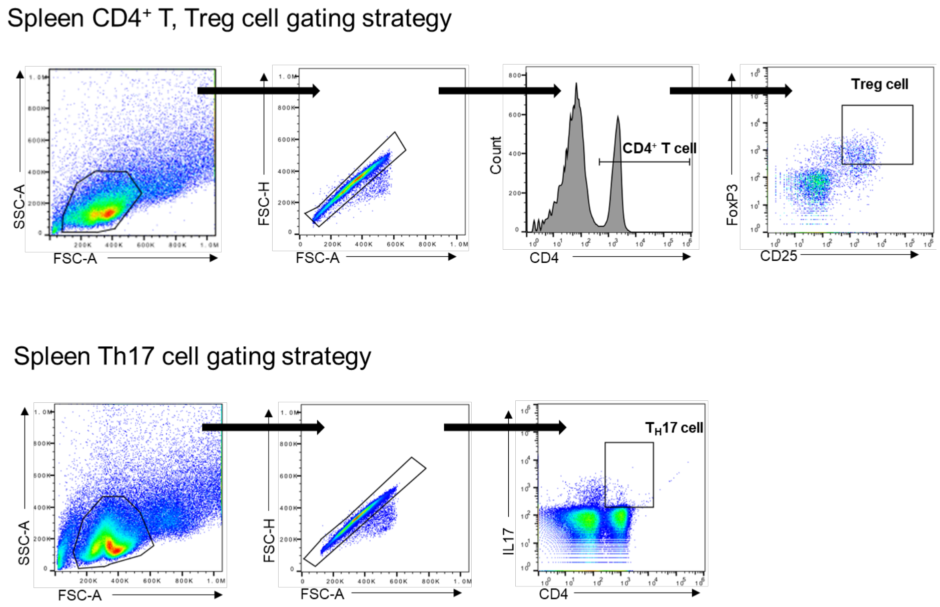

Supplementary Figure 15. Spleen CD4$^+$ T, Treg, Th17 cells FACs gating strategy.

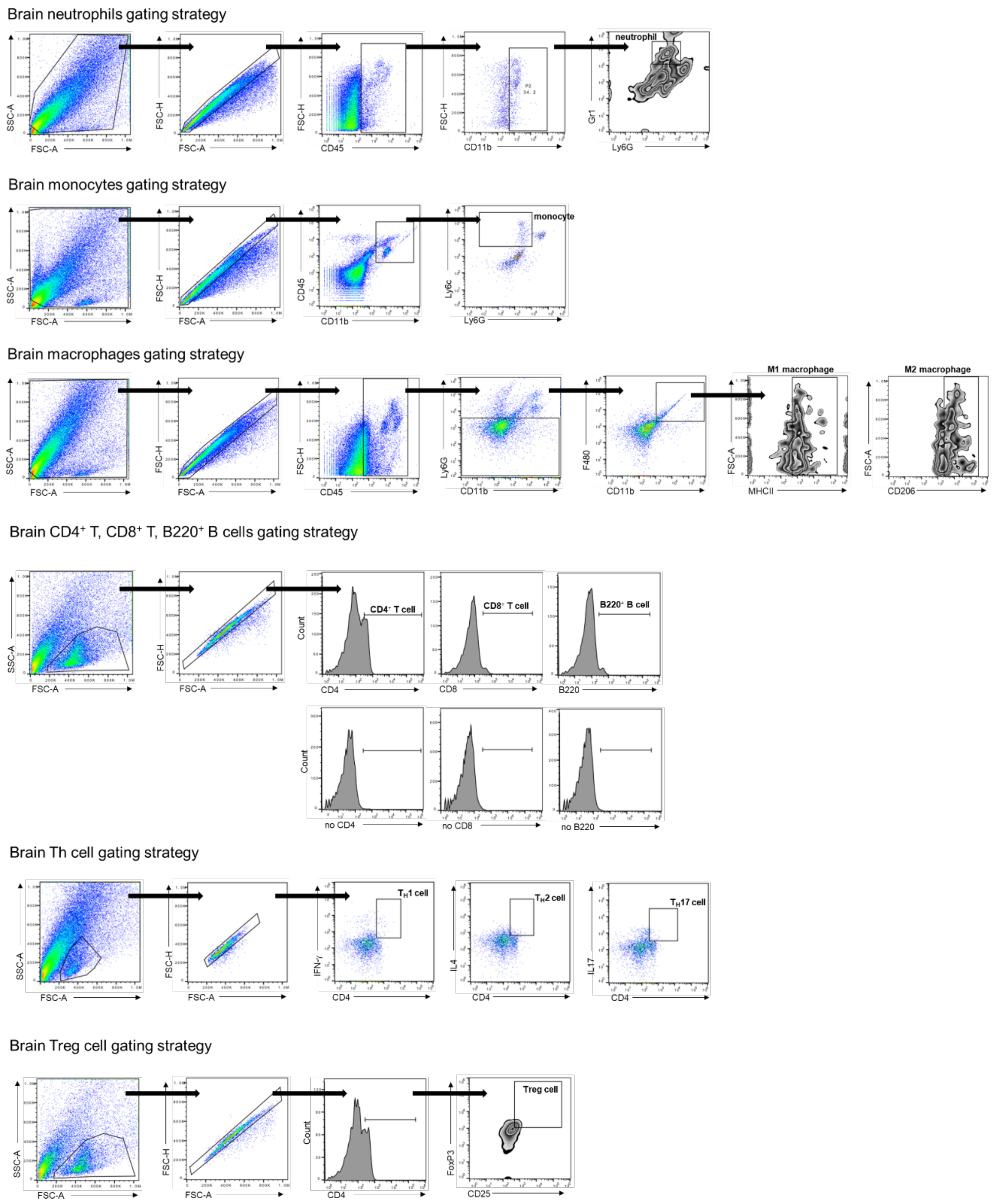

Supplementary Figure 16. Brain immune cells FACs gating strategy.

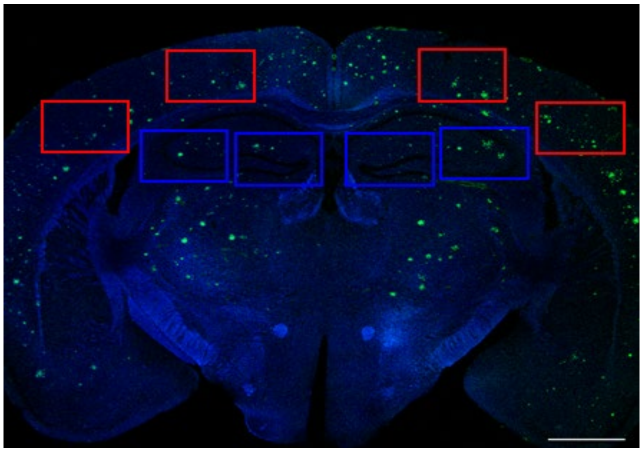

Supplementary Figure 17. Schematic depicting for quantification of immunostaining in cortex and hippocampus. Four images of 20x or 40x magnification within each region were obtained to quantification of immunostaining in this study. Red box: cortex region, Blue box: hippocampus region.

Supplementary Table 1. Human plasma information for control subjects and subjects with AD.

Clinical diagnosis	Number	Age	Sex (Female %)	Amyloid PET
Normal	15	>60	33.3	1 (normal)
MCI due to AD	15	>60	60.0	1.5 (borderline- moderate)
AD, early stage	15	>60	53.3	2-3 (moderate-severe)
AD, advanced stage	15	>60	60.0	3 (severe)

MCI, mild cognitive impairment; Amyloid PET, amyloid positron emission tomography

Supplementary Table 2. Sequences of Real-time PCR primer pairs

Gene	Forward	Reverse
mTnf-α	5'-GATTATGGCTCAGGGTCCAA-3'	5'-GCTCCAGTGAATTCGGAAAG-3'
mIl-1β	5'-CCCAAGCAATACCCAAAGAA-3'	5'-GCTTGTGCTCTGCTTGTGAG-3'
mIl-6	5'-CCGGAGAGGAGACTTCACAG-3'	5'-TTGCCATTGCACAACTCTTT-3'
miNos	5'-CACCTGGAACAGCACTCTCT-3'	5'-CTTTGTGCGAAGTGTCAGTG-3'
mIl-10	5'-AAGGCCATGAATGAATTTGA-3'	5'-TTCGGAGAGAGGTACAAACG-3'
mIl-4	5'-ATCCATTTGCATGATGCTCT-3'	5'-GAGCTGCAGAGACTCTTTCG-3'
mTgf-β	5'-TTACCTGGATGGAAGTGGAA-3'	5'-TGTTATGAGGAAGGGGACAA-3'
mArg1	5'-AAGCCAAGGTTAAAGCCACT-3'	5'-CGATTCACCTGAGCTTTGAT-3'
mIl17a	5'-CCTCAGACTACCTCAACCG-3'	5'-CTCCCTCTTCAGGACCAG-3'
mIl7f	5'-TGCTACTGTTGATGTTGGGAC-3'	5'-AATGCCCTGGTTTTGGTTGAA-3'
mRoRγt	5'-AATGGAAGTCGTCCTAGTCAG-3'	5'-CCGTGTAGAGGGCAATCTCA-3'
mRorc	5'-TGCAAGACTCATCGACAAGGC-3'	5'-AGCTTTTCCACATGTTGGCTG-3'
mCcr6	5'-GCTGTTCTTTGGGTTCTTCA-3'	5'-GAGGAGGACCATGTTGTGAG-3'
mCcl20	5'-TCCTTCCAGAGCTATTGTGG-3'	5'-GCGCACACAGATTTTCTTTT-3'
mCsf2	5'-CTCACTGGCCCCATGTATAG-3'	5'-CCTCAGGACCTTAGCCTTTC-3'
mIl-23r	5'-TTTTTCAGATGGGCATGAAT-3'	5'-CAAATCCGAGCTGTTGTTCT-3'
mIl-2	5'-CCCTTGCTAATCACTCCTCA-3'	5'-TCCGCTGTAGAGCTTGAAGT-3'
mIl-9	5'-ACCAGCTGCTTGTGTCTCTC-3'	5'-ACGGACACGTGATGTTCTTT-3'
mZo1	5'-TCTGTGCTCACCAGAGTCAA-3'	5'-AGAGAAGCACCTGGAGGTTT-3'
mClaudin5	5'-CCAGAGGGACGTGTTAAAAA-3'	5'-ACCCCTCTAAGGCTCTGGTA-3'
mOccludin	5'-TCGTGGCTTTTGCTTTAATC-3'	5'-TGCCTGCAGACACATTTTTA-3'
mGapdh	*5'-TGAATACGGCTACAGCAACA-3'*	*5'-AGGCCCCTCCTGTTATTATG-3'*

Tnf-α, tumor necrosis factor-alpha; Il-1β, interleukin 1 beta; Il6, interleukin 6; *iNos*, inducible nitric oxide synthase; *Il-10*, interleukin 10; *Il -4*, interleukin 4; *Tgf-β*, transforming growth factor beta; *Arg1*, arginase 1; *Il17a*, interleukin 17a; *Il17f*, interleukin 17f; *RoRγt, r*etineic-acid-receptor-related orphan nuclear receptor gamma t; *RoRc, r*etineic-acid-receptor-related orphan nuclear receptor c; *Ccr6,* C-C motif chemokine receptor 6; *Ccl20*, C-C motif chemokine ligand 20; *Csf2*, colony stimulating factor 2; *Il23r,* interleukin 23 receptor; *Il-2*, interleukin 2; *Il-9*, interleukin 9; *Zo-1*, Zonula occludens-1; *Gapdh*, glyceraldehyde 3-phosphate dehydrogenase.

Uncropped blots for Supplementary Figure 2i.

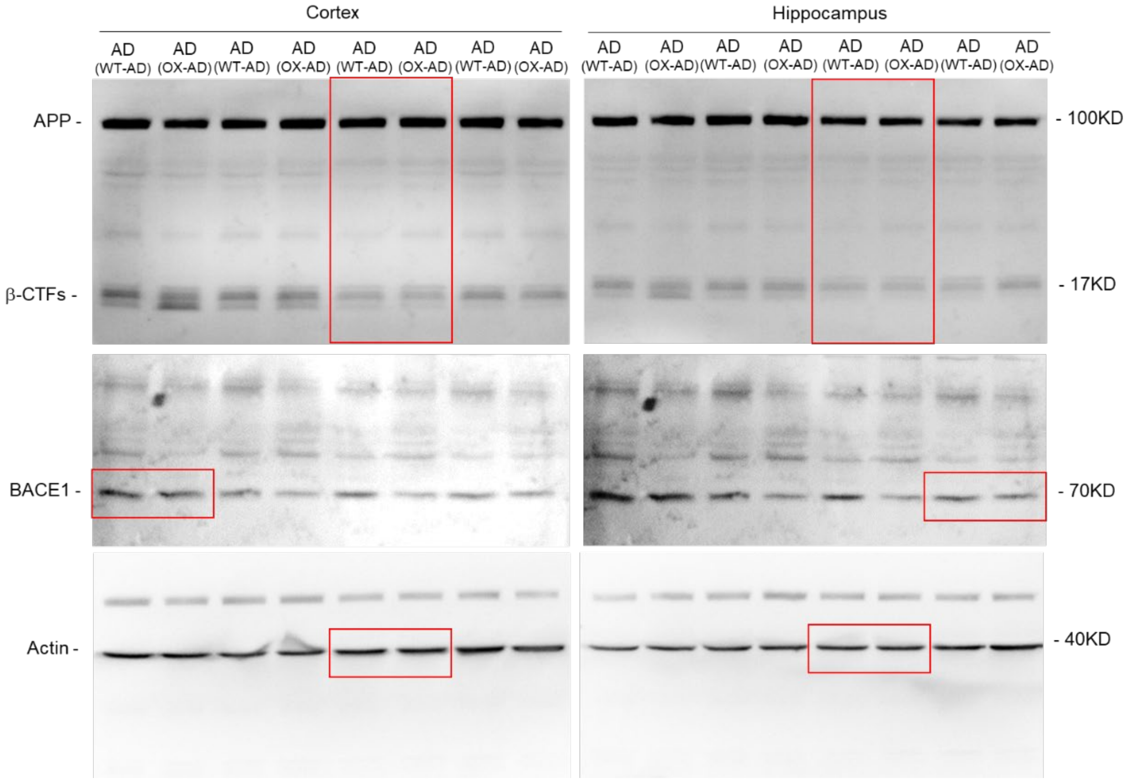

Uncropped blots for Supplementary Figure 4e.

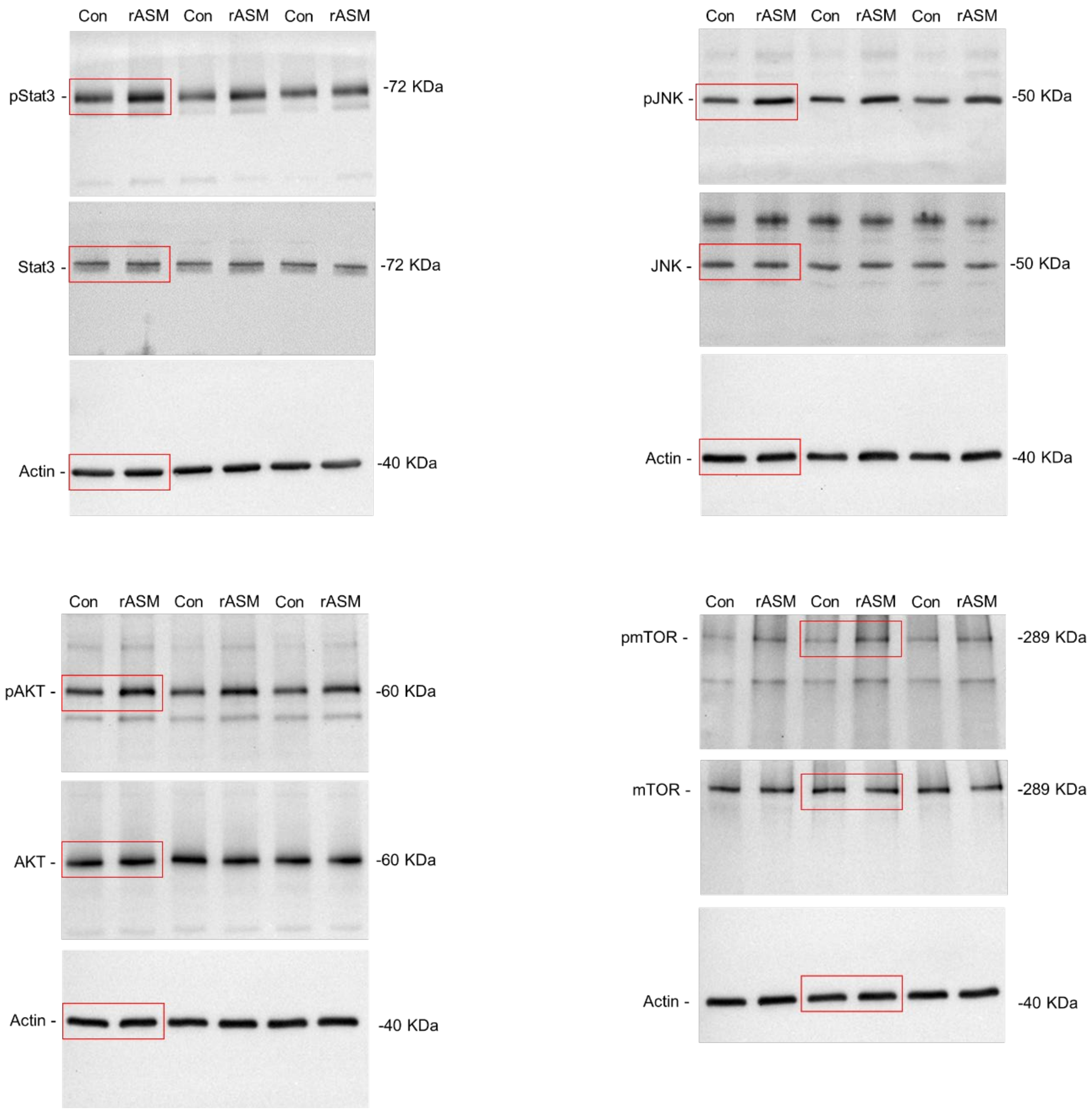

Uncropped blots for Supplementary Figure 6e and 6g.

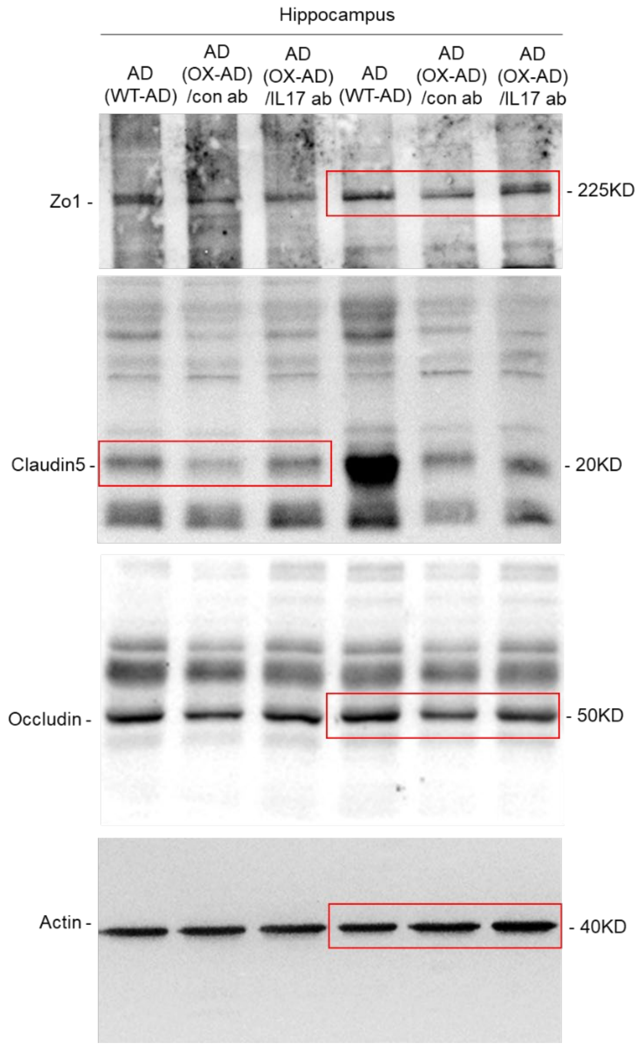

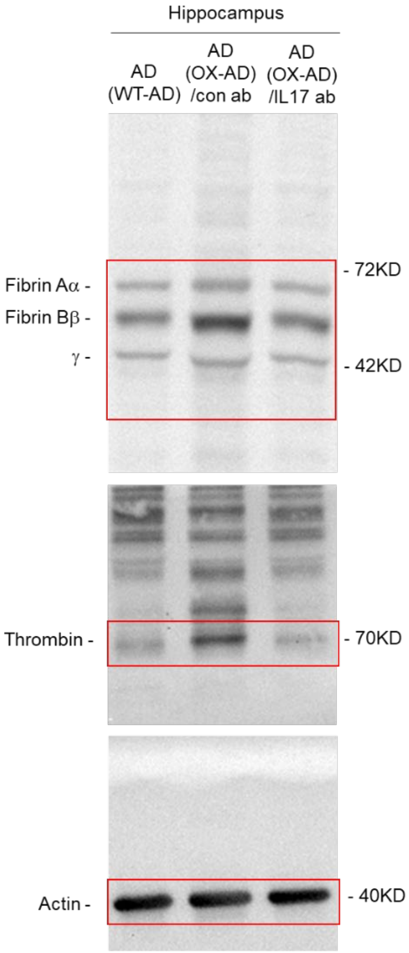

Uncropped blots for Supplementary Figure 8a and 8c.

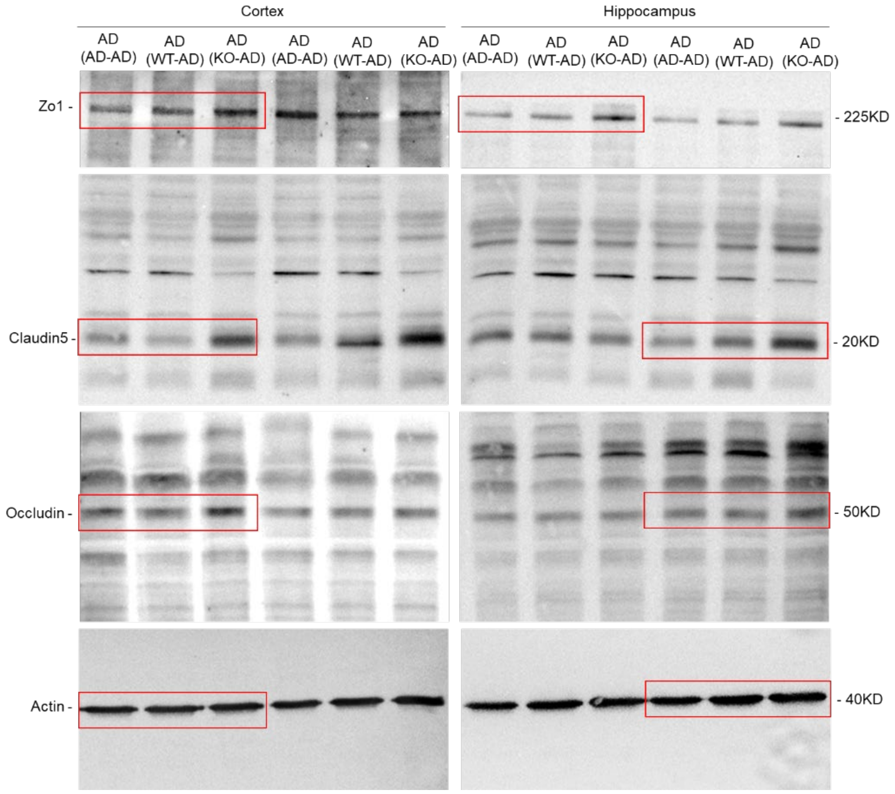

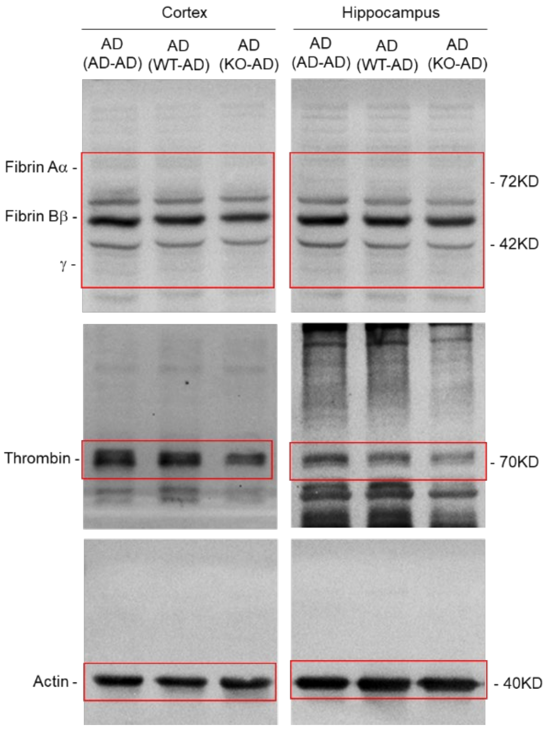

Uncropped blots for Supplementary Figure 10e.

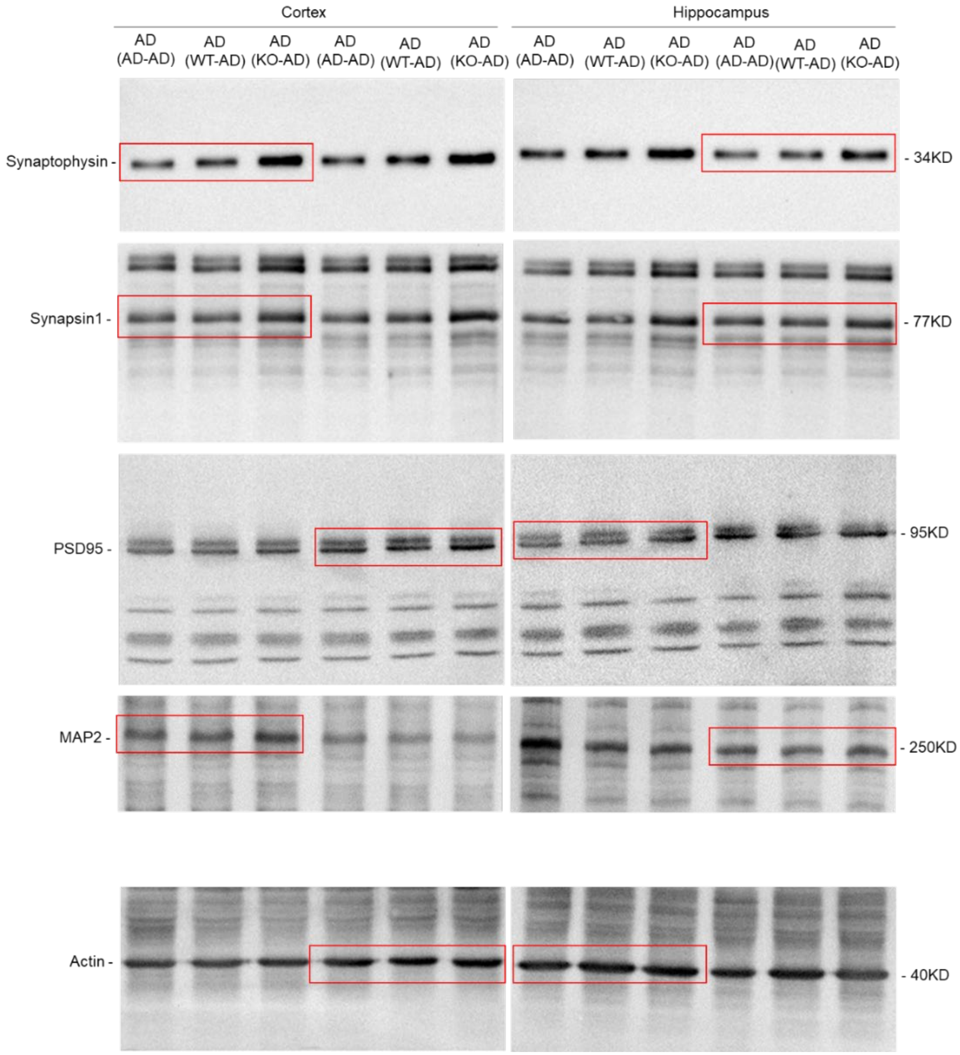

Uncropped blots for Supplementary Figure 11a and 11c.

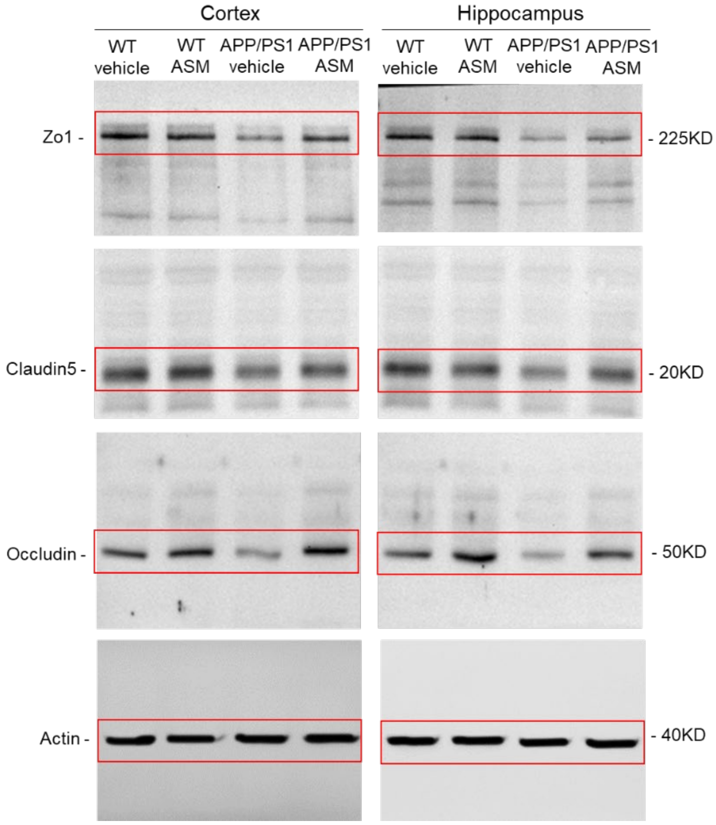

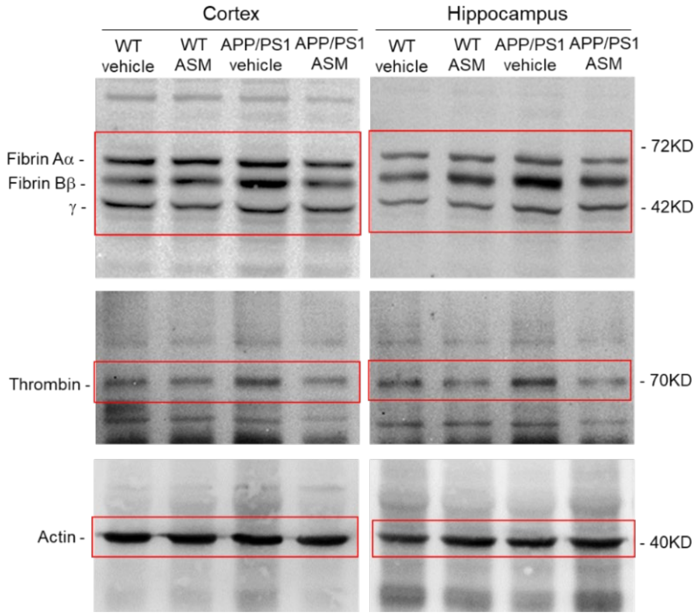

Uncropped blots for Supplementary Figure 12i.

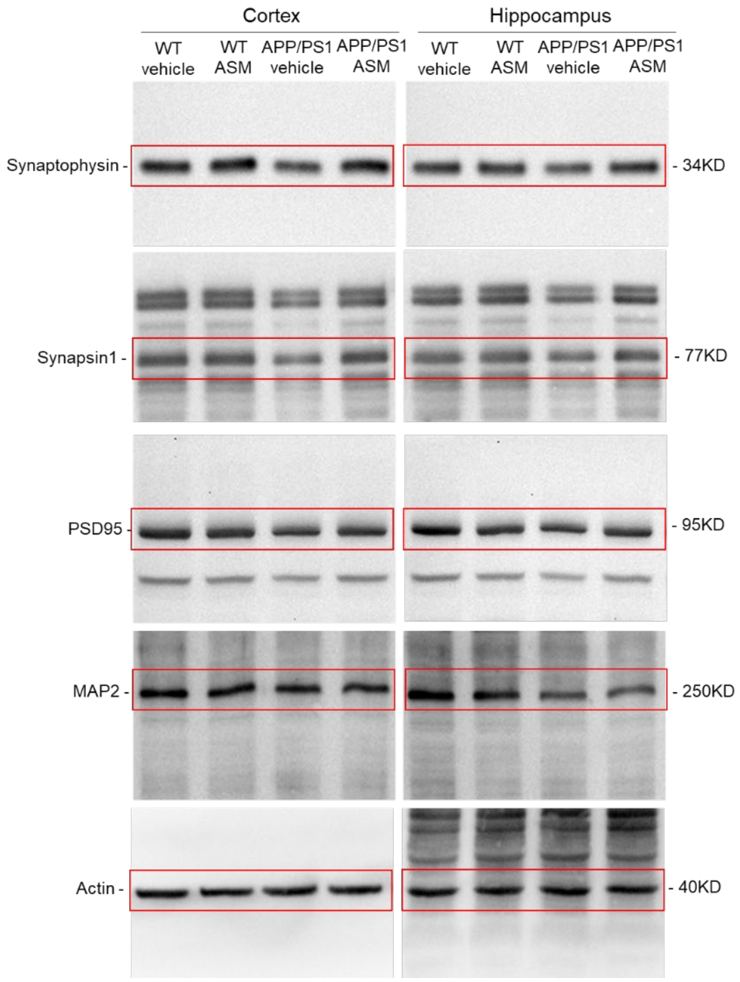

Supplement: Supplementary file 1 — Supplementary Information [file 41467_2023_37316_MOESM1_ESM.pdf]
