# Supplementary material for: Immunotherapy targeting plasma ASM is protective in a mouse model of Alzheimer’s disease
Source: Nat Commun. 2023 Mar 24;14:1631. doi: 10.1038/s41467-023-37316-z (PMC10036484; doi:10.1038/s41467-023-37316-z)
Supplement: Supplementary file 3 — Reporting Summary [file 41467_2023_37316_MOESM3_ESM.pdf]

nature portfolio

Corresponding author(s): Jae-sung Bae, Hee Kyung Jin

Last updated by author(s): Feb 23, 2023

Reporting Summary

Nature Portfolio wishes to improve the reproducibility of the work that we publish. This form provides structure for consistency and transparency in reporting. For further information on Nature Portfolio policies, see our Editorial Policies and the Editorial Policy Checklist.

Statistics

For all statistical analyses, confirm that the following items are present in the figure legend, table legend, main text, or Methods section.

n/a	Confirmed	
[ ]	[x]	The exact sample size (*n*) for each experimental group/condition, given as a discrete number and unit of measurement
[ ]	[x]	A statement on whether measurements were taken from distinct samples or whether the same sample was measured repeatedly
[ ]	[x]	The statistical test(s) used AND whether they are one- or two-sided<br>*Only common tests should be described solely by name; describe more complex techniques in the Methods section.*
[x]	[ ]	A description of all covariates tested
[ ]	[x]	A description of any assumptions or corrections, such as tests of normality and adjustment for multiple comparisons
[ ]	[x]	A full description of the statistical parameters including central tendency (e.g. means) or other basic estimates (e.g. regression coefficient) AND variation (e.g. standard deviation) or associated estimates of uncertainty (e.g. confidence intervals)
[ ]	[x]	For null hypothesis testing, the test statistic (e.g. *F*, *t*, *r*) with confidence intervals, effect sizes, degrees of freedom and *P* value noted<br>*Give P values as exact values whenever suitable.*
[x]	[ ]	For Bayesian analysis, information on the choice of priors and Markov chain Monte Carlo settings
[x]	[ ]	For hierarchical and complex designs, identification of the appropriate level for tests and full reporting of outcomes
[ ]	[x]	Estimates of effect sizes (e.g. Cohen's *d*, Pearson's *r*), indicating how they were calculated

Our web collection on statistics for biologists contains articles on many of the points above.

Software and code

Policy information about availability of computer code

Data collection	The numbers of samples used to result in statistically significant differences was calculated using G-power software (ver 3.1.9.4) with a significance level of 0.05 and a power of 0.8.
Data analysis	All software used for analysis is commercially available. For ASM activity and ceramide quantification, Waters Millennium software was used. For histologcal analysis, MetaMorph software (Molecular Devices) or IMARIS software (Bitplane) were used. For densitometric quantification of western blot, ImageJ software (National Institutes of Health) was used. For ASM IC50 determination and bindind assay, GraphPad Prism program (ver 7.0) was used. For SPR analysis, Biacore T200 Evaluation Software was used. All statistical data were graphed and comparisons between two groups were performed with two-tailed student's t-test. In cases where more than two groups were compared to each other, a one way analysis of variance (ANOVA) was used, followed by Tukey's HSD test. All statistical analysis was performed GraphPad Prism 7.0 software. This programs provide whether our data come from normal distribution. We checked the normal distribution using Shaprio-Wilk analysis. When the data existed in normal distribution, we progress further analysis. This programs also provides the equality of variances.

For manuscripts utilizing custom algorithms or software that are central to the research but not yet described in published literature, software must be made available to editors and reviewers. We strongly encourage code deposition in a community repository (e.g. GitHub). See the Nature Portfolio guidelines for submitting code & software for further information.

Data

Policy information about availability of data

All manuscripts must include a data availability statement. This statement should provide the following information, where applicable:

- Accession codes, unique identifiers, or web links for publicly available datasets
- A description of any restrictions on data availability
- For clinical datasets or third party data, please ensure that the statement adheres to our policy

All data supporting the findings of this study are available in the article and its supplementary information. Source data are provided with this paper as Source Data file.

Human research participants

Policy information about studies involving human research participants and Sex and Gender in Research.

Reporting on sex and gender	Human plasma samples were obtained from both sexes with AD and age-matched non-AD controls.
Population characteristics	Normal subjects: Amyloid PET 1 (normal), age>60, number=15, Female %=33.3<br>MCI due to AD: Amyloid PET 1.5 (borderline-moderate), age>60, number=15, Female %=60<br>Early AD: Amyloid PET 2-3 (moderate-severe), age>60, number=15, Female %=53.3<br>Advanced AD: Amyloid PET 3 (severe), age>60, number=15, Female %=60
Recruitment	All participants were recruited from Hanyang University Hospital (Seoul, South Korea). A neurologist specialist examined and confirmed diagnosis of AD patients.
Ethics oversight	Informed consent was obtained from all subjects according to the ethics committee guidelines at the Hanyang University Hospital (IRB no. HYUH 2016-12-029-003).

Note that full information on the approval of the study protocol must also be provided in the manuscript.

Field-specific reporting

Please select the one below that is the best fit for your research. If you are not sure, read the appropriate sections before making your selection.

☒ Life sciences ☐ Behavioural & social sciences ☐ Ecological, evolutionary & environmental sciences

For a reference copy of the document with all sections, see nature.com/documents/nr-reporting-summary-flat.pdf

Life sciences study design

All studies must disclose on these points even when the disclosure is negative.

Sample size	The numbers of samples used to result in statistically significant differences was calculated using G-power software (ver 3.1.9.4) with a significance level of 0.05 and a power of 0.8.
Data exclusions	We did not exclude the samples or animals except when the mice were dead.
Replication	All experiments were performed in this manuscript at least three times independent biological replicates. All attempt to reproduce the results were successful.
Randomization	We used block randomization method to allocate the samples/animals to experimental groups. This method is usually used to ensure a balance in sample size. For example, when two treatment group was tested, the block size is four (2 x 2= 4). Possible treatment allocations within each block are (1) AABB, (2) BBAA, (3) ABAB, (4) BABA, (5) ABBA, (6) BAAB. We selected the block size depended on the number of treatments. The block size was short enough to prevent imbalance, and long enough to prevent guessing allocation in trials. It was at least 2x number of treatments.
Blinding	We were blinded some process of experimental progress such as data collection and data analysis. Experimenters were blinded to the identity of experimental groups until the end of data collection and analysis for at least one of the independent experiments.

Reporting for specific materials, systems and methods

We require information from authors about some types of materials, experimental systems and methods used in many studies. Here, indicate whether each material, system or method listed is relevant to your study. If you are not sure if a list item applies to your research, read the appropriate section before selecting a response.

Materials & experimental systems

n/a	Involved in the study
☐	☒ Antibodies
☐	☒ Eukaryotic cell lines
☒	☐ Palaeontology and archaeology
☐	☒ Animals and other organisms
☒	☐ Clinical data
☒	☐ Dual use research of concern

Methods

n/a	Involved in the study
☒	☐ ChIP-seq
☐	☒ Flow cytometry
☒	☐ MRI-based neuroimaging

Antibodies

Antibodies used

The profiles of antibodies for flow cytometry were as follows: mouse anti-CD11b APC (1:100, BD Bioscience, 553312), mouse anti-CD115 PE (1:100, Thermo Fisher Scientific, 12-1152-82), mouse anti-Ly6C FITC (1:100, BD Bioscience, 553104), mouse anti-Ly6G APC-Cy7 (1:100, BD Bioscience, 557661), mouse anti-Ly6G FITC (1:100, BD Bioscience, 551460), mouse anti-lineage biotin (1:10, Miltenyl Biotec, 130-090-858), anti-biotin streptavidin PB (1:100, Invitrogen, S11222), mouse anti-CD11b PE (1:100, BD Bioscience, 557397), mouse anti-F4/80 APC (1:100, Thermo Fisher Scientific, 14-4801-82), mouse anti-CD45 PerCp Cy5.5 (1:100, BD Bioscience, 550994), mouse anti-CD11b PE (1:100, BD Bioscience, 557397), mouse anti-CD11b PeCy5 (1:100, Tonbo Bioscience, 55-011), mouse anti-CD45 APC-Cy7 (1:100, BD Bioscience, 557659), mouse anti-MHCII PE (1:100, BD Bioscience, 557000), mouse anti-CD206 PE-Cy7 (1:100, Thermo Fisher Scientific, 25-2061-82), mouse anti-CD4 FITC (1:100, BD Bioscience, 553047), mouse anti-CD8 APC (1:100, eBioscience, 17-0081-82), mouse anti-CD25 PE (1:100, eBioscience, 12-0251-82), mouse anti-FoxP3 APC (1:100, eBioscience, 17-5773-82), mouse anti-B220 PE (1:100, Tonbo Bioscience, 50-0452), anti-IFN-gamma APC (1:100, BD Bioscience, 553047), mouse anti-IL4 PE (1:100, Bio Legend, 504104), and mouse anti-IL17 PerCp Cy5.5 (1:100, BD Bioscience, 553142)

The profiles of antibodies for immunofluorescence staining were as follows: 6E10 (mouse, 1:100, Signet, SIG39300), SMA (mouse, 1:400, Sigma-Aldrich, A2547), Iba1 (rabbit, 1:500, Wako, 019-19941), GFAP (rabbit, 1:500, Dako, N1506), Lamp1 (mouse, 1:200, Abcam, ab24170), Fibrinogen (Fibrin, rabbit, 1:500, Dako, A008002), fluorescein labeled L. esculentum lectin (1:100, Vector Laboratories, FL-1171), CD31 (goat, 1:100, R&D system, AF3628), Collagen IV (rabbit, 1:100, Abcam, ab6586), Aquaporin-4 (chicken, 1:100, Synaptic Systems, 429006), GFP (rat, 1:1000, Abcam, ab13970), and anti-RORγ (1:100, Rabbit, Abcam, ab207082) antibody.

The profiles of antibodies for western blotting were as follows: APP (mouse, 1:500, Signet, SIG39300), BACE-1 (mouse, 1:1,000, Millipore, MAB5308), Synaptophysin (rabbit, 1:2000, Abcam, ab32127), PSD95 (mouse, 1:1000, Millipore, MAB1596), Synapsin 1 (rabbit, 1:1000, Synaptic systems, 106103), MAP2 (chicken, 1:10000, Abcam, ab5392), p-Stat3 (rabbit, 1:1000, Cell Signaling Technology, 9145), Stat3 (mouse, 1:1000, Cell Signaling Technology, 9139), p-JNK (mouse, 1:1000, Cell Signaling Technology, 9255), JNK (rabbit, 1:1000, Cell Signaling Technology, 9252), p-Akt (rabbit, 1:1000, Cell Signaling Technologies, 4060), Akt (rabbit, 1:1000, Cell Signaling Technologies, 4091), p-mTOR (rabbit, 1:1000, Cell Signaling Technology, 5536), mTOR (rabbit, 1:1000, Cell Signaling Technology, 2983), Fibrinogen (Fibrin, rabbit, 1:500, Dako, A008002), Thrombin (goat, 1:100, Santa Cruz Biotechnology, sc-23355), ZO-1 (rabbit, 1:500, Invitrogen, 40-2200), Occludin (mouse, 1:500, Invitrogen, 33-1500), Claudin5 (mouse, 1:500, Invitrogen, 35-2500), beta-actin (1:1,000, Santa Cruz, SC-1615), Rabbit-HRP (1:1000, Cell signaling, 7074), goat-HRP (1:1000, Santa Cruz Biotechnology, sc2020) and mouse-HRP (1:1000, Cell signaling, 7076).

Monoclonal ASM antibody generation: The synthesis of recombinant human ASM (rASM) and production of mouse monoclonal antibodies were performed by Genscript. Screening of 110 hybridomas was performed by ELISA against rASM. Positive clones were expanded and re-tested to confirm epitope reactivity to rASM. ASM antibody (23A21C3) was used for in vitro and in vivo treatment.

Validation

All antibodies were validated by the manufacturer or publications (Park et al. 2018, Neuron; Park et al. 2022, PNAS; Scholz et al. 2016, EMBO Mol. Med; Park et al. 2016, Stem cells; Aryal et al. 2016, Nat. Commun; Möhle et al. 2016, Cell Rep; Zenaro et al. 2015, Nat. Med; Yang et al. 2011, Nat Immunol; Knier et al. 2018, Nat Immunol; Zhang et al. 2013, Plos One).

Eukaryotic cell lines

Policy information about cell lines and Sex and Gender in Research

Cell line source(s)	BV2 microglial cells were purchased from Accegen (ABC-TC212S). Expi293F cells were purchased from Thermo Fisher Scientific (A14527).
Authentication	Cell line authentiation was not performed.
Mycoplasma contamination	Cells were tested to confirm absence of mycoplasma contamination using MycoAlert PLUS Mycoplasma detection kit (Lonza, LT07).
Commonly misidentified lines (See ICLAC register)	No commonly misidentified cell lines were used in this study.

Animals and other research organisms

Policy information about studies involving animals; ARRIVE guidelines recommended for reporting animal research, and Sex and Gender in Research

Laboratory animals	3-, 6-, or 9-month-old C57BL/6 wild type (WT) mice (The Jackson Laboratory), 3-month-old Tie2-Cre mice (stock number 008863, The Jackson Laboratory), loxP-flanked Tg-Smpdstop mice (Smpd1ox/ox mice, C57BL/6 background), 3- or 7.5-month-old Smpd1-/- mice (C57BL/6 background), and 3-month-old Il17a-EGFP knockin mice (stock number 018472, The Jackson Laboratory). To obtain endothelial cell-specific ASM overexpressing mice, Tg-Smpdstop mice were crossed with Tie2-Cre mice. Transgenic mouse lines over-expressing the hAPP695swe (APP) and presenilin-1M146V (PS1) mutations were originated from GlaxoSmithKline (Harlow, UK). We used littermate mice that were sex- and age-matched between experimental groups. Both male and female mice were used for all experiments except behavioral studies using male mice. Mice were housed at a 12 h day/12 h night cycle, 21-22̊C and 50-60 % humidity with free access to water and food pellets.<br><br>To examine the possible prophylactic effects of plasma ASM-targeting active immunotherapy, 50 µg/100 µl of ASM protein or 100 µl PBS was mixed with 100 µl of the complete freund's adjuvant and repeatedly passed through a micro-emulsifying needle (Cadence Science, CAD7977) until the mixture became pasty. Then, 200 µl of mixture was injected intraperitoneally (i.p.) into 6-mo-old WT and APP/PS1 mice. Second and third immunization was performed every 2 weeks with 25 µg/50 µl of ASM protein or 50 µl PBS mixed with 50 µl of the incomplete freund's adjuvant. Four weeks after third immunization, mice were immunized 25 µg/50 µl of ASM protein or 50 µl PBS mixed with 50 µl of the incomplete freund's adjuvant. After 4 weeks, mice were sacrificed for analysis. For passive immunotherapy, IgG isotype control antibody (50 mg kg-1, R&D system, MAB002) or ASM antibody (50 mg/kg, 23A12C3) was injected twice a week i.p. for 8 weeks to 7-mo-old APP/PS1 mice until the age of 9 months.
Wild animals	No wild animals were used in the study.
Reporting on sex	Both male and female mice were used for all experiments except behavioral studies using male mice.
Field-collected samples	No field-collected samples were used in the study.
Ethics oversight	Animal experiments were conducted in accordance with the protocols approved by the Institutional Animal Care and Use Committee (IACUC) of Kyungpook National University

Note that full information on the approval of the study protocol must also be provided in the manuscript.

Flow Cytometry

Plots

Confirm that:

- [x] The axis labels state the marker and fluorochrome used (e.g. CD4-FITC).
- [x] The axis scales are clearly visible. Include numbers along axes only for bottom left plot of group (a 'group' is an analysis of identical markers).
- [x] All plots are contour plots with outliers or pseudocolor plots.
- [x] A numerical value for number of cells or percentage (with statistics) is provided.

Methodology

Sample preparation	Brain was dissected and immediately transferred in ice-cold HBSS (Gibco). After gentle mincing, the brain was digested in a HBSS solution containing collagenase P (0.2 mg ml-1, Roche), dispase II (0.8 mg ml-1, Roche), DNase I (0.01 mg ml-1, Roche), and collagenase A (0.3 mg ml-1, Roche) at 37 °C for 1h under gentle rocking. Digestion was stopped by adding FBS (Gibco) on ice. The supernatants were centrifuged at 250 g for 10 min at 4 °C. The pellet was resuspended in 25 % BSA (Gibco)/PBS (Gibco) for myelin removal. Following a centrifugation step at 3,000 g for 30 min at 4 °C, the myelin containing supernatant was discarded. The cell pellets were then resuspended in 1 ml of HBSS and filtered through a 40m mesh, followed by a washing step in HBSS. The cell pellets were resuspended in 1 ml of red blood cell lysis buffer (BD Biosciences) and incubated at RT for 10 min for lysis of erythrocytes. Subsequently, 2 ml of MACs buffer (Miltenyl Biotec, 130-091-222) was added and centrifuged at 250 g for 10 min at 4 °C. Blood cells were prepared as previously described with minor modifications57. To obtain peripheral blood mononuclear cells (PBMCs), blood was collected in sodium-heparin tube (BD Biosciences, 367871) by cardiac puncture and blood was gently layered in the top of histopaque (Sigma-Aldrich, 10771). After centrifuge (400×g, 30 min), PBMCs formed in the interphase between histopaque and plasma were collected and washed once with PBS. For analysis of myeloid immune cells, red blood cells were lysed once at 4 °C for 10 min in 0.15 M NH4Cl (STEMCELL Technologies) and washed once with PBS.
Instrument	The fluorescence data were acquired on a Attune NxT flow cytometer (Thermo Fisher)
Software	Attune NxT software (Thermo Fisher) was used to collect the data.<br>FlowJo analytical software (Tree Star, Inc.) was used to analyze the data.
Cell population abundance	Cell sorting was not performed. At least 50,000-100,000 relevant events were acquired for all in vitro or in vivo flow cytometry analyses.

Gating strategy	Debris was removed by gating on main cell populations. Based on the pattern of FSC-A/SSC-A, cells in the lymphocyte or myeloid cells gate were used for analysis of T, B cell subsets or neutrophils, monocytes and macrophages.<br>Singlets were gated according to the pattern of FSC-H vs. FSC-A. A positive threshold for each antibody staining was defined compared to a negative control. The same amount of threshold was applied to all samples within on analysis.

☒ Tick this box to confirm that a figure exemplifying the gating strategy is provided in the Supplementary Information.
